# Ru^δ+^-Engineered La_2_Ti_2–*x*_Ru_*x*_O_7_ Perovskite with Dynamic Lattice Oxygen Vacancies for Efficient and Stable Photothermal Dry Reforming of Methane

**DOI:** 10.1021/acs.langmuir.6c05014

**Published:** 2026-10-01

**Authors:** Kamran Kamran, Muhammad Tahir

**Affiliations:** Chemical and Petroleum Engineering Department, 11239 United Arab Emirates University, P.O.BOX, 15551 Al Ain, United Arab Emirates

## Abstract

Dry reforming of methane (DRM) is a sustainable method to produce valuable syngas from two important greenhouse gases, CH_4_ and CO_2_. Traditional thermal DRM requires high temperatures, resulting in high carbon deposition, while photothermal catalysis reduces the energy barrier by using solar light and creates charge carriers. However, most of the photothermal catalysts have limited mobility of oxygen and poor charge separation, which limits the reaction kinetics. Pristine La_2_Ti_2_O_7_ is a layered perovskite that has a slow Ti redox activity and low oxygen-vacancy concentration, thereby restricting its catalytic activity. Ru doping at the Ti sites of La_2_Ti_2–*x*_Ru_*x*_O_7_ reconstructs the electronic structure, activating the dynamic lattice oxygen exchange. This substitution leads to the generation of more oxygen vacancies and enhances the interaction of the Ru–O–Ti interface, which results in highly active sites for the activation of CH_4_ and CO_2_ molecules. In the presence of photons, pristine La_2_Ti_2_O_7_ generated 9.3 mmol g^–1^ h^–1^ H_2_ and 12.4 mmol g^–1^ h^–1^ CO. La_2_Ti_2–*x*_Ru_*x*_O_7_, however, showed outstanding enhancement with production rates of 339.58 mmol g^–1^ h^–1^ H_2_ and 494.46 mmol g^–1^ h^–1^ CO. This is an increase of more than 35-fold and 39-fold, respectively. Mechanistic studies show that light irradiation leads to the dissociation of CO_2_, which maintains the oxidation of carbon-containing intermediates using activated lattice oxygen by the Mars–van Krevelen pathway. The presence of Ru^δ+^ species and mobile oxygen in the lattice provides continuous activation of the active sites. This redox synergy is a continuous suppression of carbon deposition, as well as provides excellent catalytic stability. It sets the framework for a promising pathway for the sustainable synthesis of syngas with high activity, selectivity, and stability.

## Graphical abstract

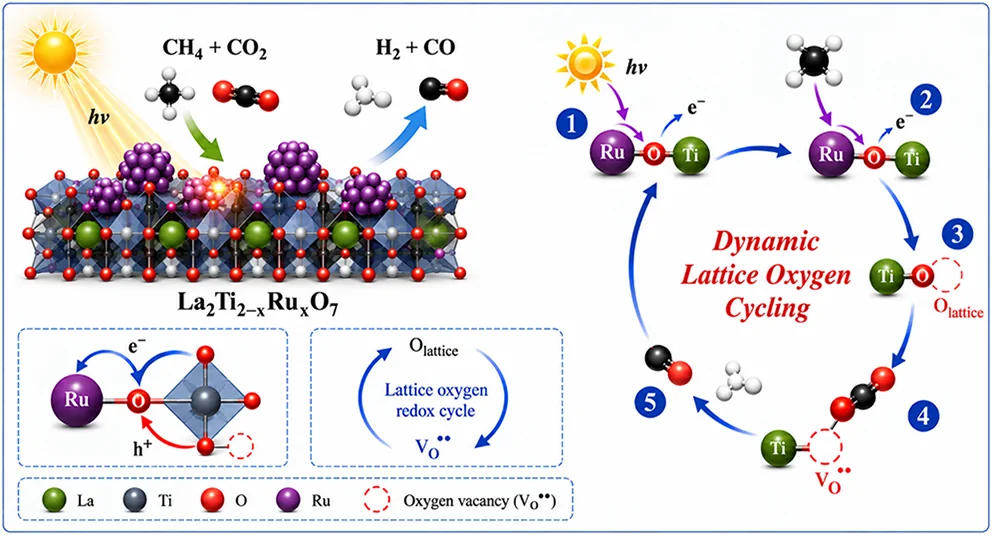

## Introduction

As climate change and energy sustainability problems have emerged, technologies related to the use of greenhouse gases and the production of useful fuels have become more interesting.^1^ Dry reforming of methane (DRM) is a viable process that can convert CH_4_ and CO_2_ to synthesis gas.^2^ DRM is intrinsically very endothermic (Δ*H*°_298_ = +247 kJ mol^–1^) and must conventionally be conducted at temperatures above 700 °C under thermocatalytic conditions. These extreme conditions can easily lead to catalyst deactivation due to nanoparticle sintering and carbon deposition.^3^ To overcome these challenges, photothermal catalysis has become an attractive strategy. It integrates energy harvesting from the sun and thermal catalysis to achieve efficient activation of the reactants and drive DRM with lower-energy consumption.^4^ Thus, the development of advanced DRM catalysts with good photothermal properties has been gaining research interest recently.^5^

The excellent thermal stability, tunable electronic properties, and flexible crystal structure of perovskite oxides make them potential multifunctional catalyst materials and have received much attention.^6^ Perovskite oxides are known to allow for a wide variety of cation substitutions which can provide precise control over lattice defects, oxygen vacancies, redox behavior, and electronic conductivity.^7^ The lattice oxygen in perovskite oxides can directly participate in the activation of CH_4_ by a Mars–van Krevelen pathway.^8^ Lattice oxygen may also be active in removing carbon species at the surface and in regenerating oxygen vacancies by the activation of CO_2_.^9^ Furthermore, perovskite structures provide strong metal–support interactions that can stabilize highly dispersed metal species, while enhancing the durability of the catalyst and preventing nanoparticle sintering during DRM.^10^ Li et al.^11^ showed that Mn-doped LaFeO_3_ has an appreciable activation of the lattice oxygen to enhance the activity of photothermal DRM and coke resistance. To realize Ru/LaFeO_3_ for photothermal DRM, Li et al.^12^ designed a photothermal catalytic system comprising Ru and LaFeO_3_ and found that the synergistic Ru–LaFeO_3_ interaction enhances the activation of methane molecules and provides increased participation of lattice oxygen. The perovskite SrTiO_3_ has been proven by Yang et al.^13^ as a support for photothermal DRM, and it has been shown that CH_4_ and CO_2_ activation can be enhanced by Ru–Au synergy.

La_2_Ti_2_O_7_ is an attractive structural platform among Ti-based perovskite-related materials due to the possibility of engineered electronic structure, defect chemistry, and surface reactivity by B-site substitutions in its layered structure.^14^ The introduction of aliovalent transition-metal species into Ti sites can create a new Ti–O structural environment and participate in the formation of oxygen vacancies, charge transport, and surface redox reactions.^15^ These properties are especially important for DRM since the efficient adsorption/activation of CO_2_ and the removal of carbonaceous intermediates are essential for maintaining the catalytic performance.^16^ Ruthenium has a particularly high ability to break the C–H bonds and is also very resistant to carbon deposition during reforming, making it one of the most effective noble metals for the activation of methane.^17^ Ru can be used to tune the electronic structure, increase oxygen mobility, and increase charge transfer and redox properties when added to perovskite lattices.^12,18^ The properties of Ru-containing perovskites render them attractive for DRM using a photothermal approach.^19^ Duan et al.^20^ reported that photoexcited electron transfer to Ru sites increases, and lattice oxygen of BaTiO_3_ enhances oxygen vacancies. Su et al.^21^ presented that electron migrates from Ru sites to BaTiO_3_ to oxidize CH* to CHO* and generate oxygen vacancies to convert CO_2_ to CO. Despite its interesting properties, La_2_Ti_2_O_7_ as a photothermal catalyst in the dry reforming reaction of methane has not been the focus of much research. The synergistic effects of Ru incorporation on the electronic structure, surface chemistry, and mechanism of photothermal DRM with La_2_Ti_2_O_7_ have not been studied systematically.

In this study, we show that Ru engineering of layered La_2_Ti_2–*x*_Ru_*x*_O_7_ is an effective strategy to alter its electronic structure and lattice oxygen chemistry to promote the photothermal dry reforming of CH_4_. Substituting Ru at Ti sites causes a change in its local Ru–O–Ti coordination environment, which helps in electronic coupling and charge redistribution, thus oxygen-vacancy formation, and oxygen mobility. The role of the Ru-associated sites is to activate CH_4_, whereas that of defect-rich Ti–O environments is to adsorb and activate CO_2_. This coupling among Ru-mediated activation of CH_4_, Ti-based lattice oxygen redox chemistry, and regeneration of oxygen vacancies allows a dynamic Mars–van Krevelen-type cycle for the oxidation of carbonaceous intermediates and CO_2_ to regenerate surface oxygen species. This cooperative approach facilitates the production of syngas and prevents carbon deposition during DRM. The present study thus establishes the relationship among Ru substitution, lattice oxygen chemistry, and the performance of DRM in the layered perovskite material La_2_Ti _2–*x*_ Ru_*x*_O_7_. This offers insights into the rational design of such layered perovskite catalysts for the efficient and stable conversion of greenhouse gas emissions.

## Experimental Section

### Materials

Lanthanum nitrate hexahydrate La­(NO_3_)_3_·6H_2_O, titanium tetraisopropoxide (TTIP) (Ti (OCH­(CH_3_)_2_)_4_), ruthenium chloride trihydrate RuCl_3_·3H_2_O, citric acid (C_6_H_8_O_7_), and ethylene glycol (CH_2_OH)_2_ were used.

### Synthesis of La_2_Ti_2_O_7_

La_2_Ti_2_O_7_ was prepared using the sol–gel method. Initially, 1.94 g of La (NO_3_)_3_·6H_2_O was dissolved in 40 mL of water, then 1.3 mL of TTIP was dissolved in 20 mL of ethanol in a separate beaker and added to the lanthanum solution with continuous stirring. C_6_H_8_O_7_ (5 g) and 1.3 mL of (CH_2_OH)_2_ were added to the lanthanum solution. The temperature was set at 60 °C, and the solution was continuously stirred until a viscous gel was achieved. This solution was dried at 120 °C for 24 h to obtain a brown dried gel, which was ground and calcined at 800 °C for 6 h to obtain La_2_Ti_2_O_7_ powder.

### Synthesis of La_2_Ti_2–*x*_Ru_*x*_O_7_

The same synthesis method was repeated for La_2_Ti_2-x_Ru_x_O_7_; however, after adding TTIP, RuCl_3_·3H_2_O was dissolved in warm water of appropriate quantities (*x* = 0.01–0.04) and poured into the lanthanum solution. After that, C_6_H_8_O_7_ and (CH_2_OH)_2_ were added in desired quantities. Thus, La_2_Ti_1.99_Ru_0.01_O_7_, La_2_Ti_1.98_Ru_0.02_O_7_, La_2_Ti_1.97_Ru_0.03_O_7_, and La_2_Ti_1.96_Ru_0.04_O_7_ were prepared. The synthesis method is illustrated in Figure 1(a).

**Figure 1:**
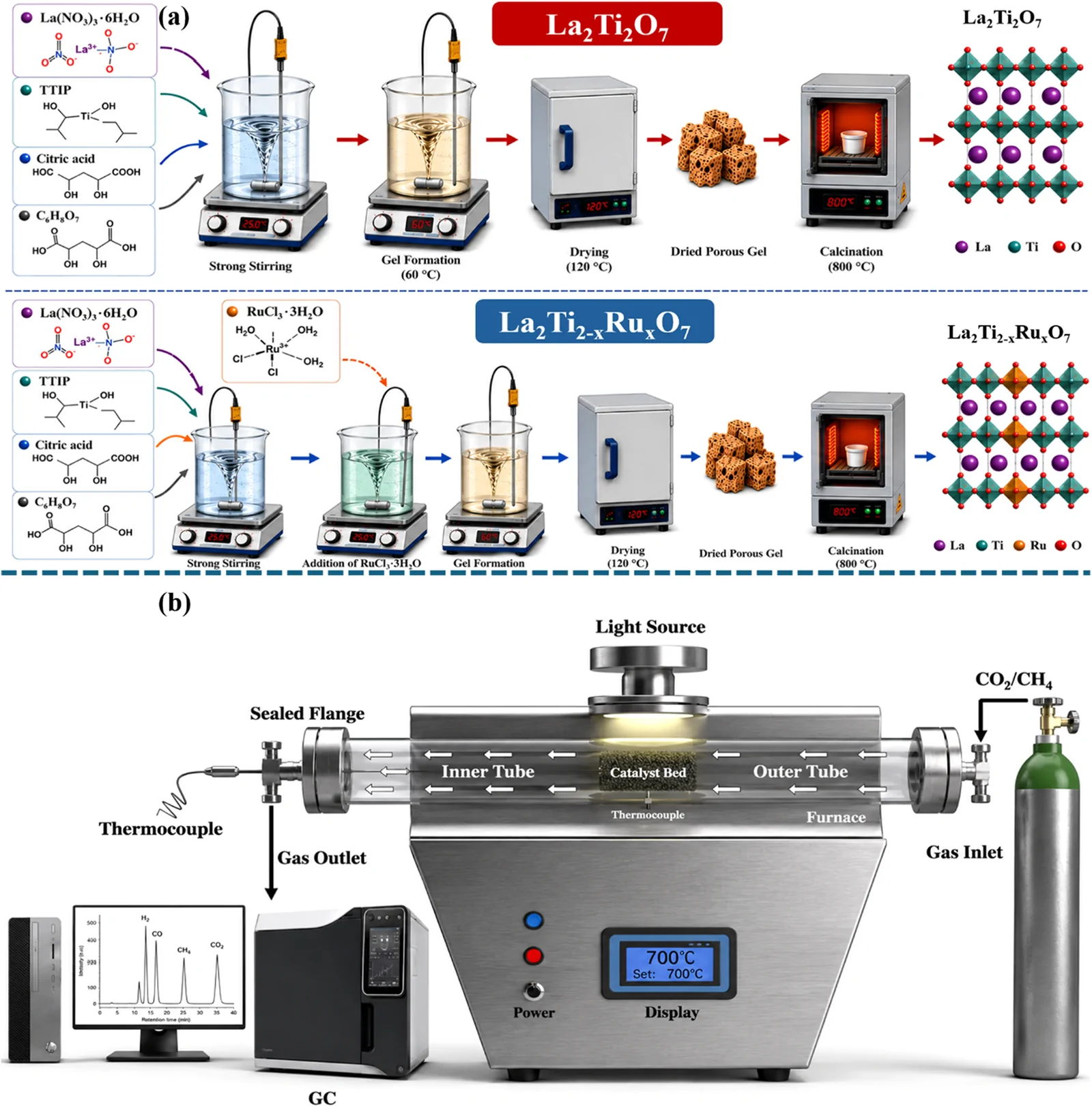
(a) Synthesis of La_2_Ti_2_O_7_ and La_2_Ti_2–*x*_Ru_*x*_O_7_ catalysts. (b) Schematics of the photothermal reactor.

### Photothermal DRM Performance Measurement

Photothermal dry reforming experiments were carried out using a specially designed double-quartz-tube reactor system, with an outer reaction tube and an inner catalyst tube. To prevent the loss of catalyst during the operation, quartz glass wool was laid down along the central axis of the catalyst bed before loading the catalyst, and the inner quartz tube was the catalyst holder, with a width of the sample bed of 3 cm and a length of 28.9 cm. About 0.1 g of the catalyst was spread uniformly on the sample holder in the center of the outer quartz tube. The outer tube served as the reaction chamber, the reactant gases passed through it, and the catalyst was irradiated directly through the quartz wall. To provide photothermal irradiation, a 300 W xenon light source was placed directly above the catalyst bed. The power density of the incident light was 2.32 W cm^–2^. The feed gas mixture was 50% CH_4_ and 50% CO_2_ fed with calibrated mass flow controller at a total flow rate of 50 mL min^–1^. Two thermocouples were used for accurate monitoring of the thermal behavior during the photothermal operation: one under the outer quartz tube to measure the reactor temperature, and another in the inner catalyst holder to measure the actual catalyst-bed temperature. A gas chromatograph (Shimadzu GC-2030) with both BID and FID detectors was used to analyze the reaction products from the reactor outlet. A schematic of the reactor is illustrated in Figure 1(b).

The conversion of CO_2_ and CH_4_ (X_CH_4__ and X_CO_2__), the rate of H_2_ and CO (r_H_2_ and_ _CO_), and the H_2_/CO ratio (R_H_2_/CO_) were calculated using eqs ([Disp-formula eq1]) to ([Disp-formula eq6]).

1 $$X_{{\mathrm{CH}}_{4}}=\frac{F_{\mathrm{in}}Y_{{\mathrm{in}}_{{\mathrm{CH}}_{4}}}-F_{\mathrm{out}}Y_{{\mathrm{out}}_{{\mathrm{CH}}_{4}}}}{F_{\mathrm{in}}Y_{{\mathrm{in}}_{{\mathrm{CH}}_{4}}}}\times 100\%$$

2 $$X_{{\mathrm{CO}}_{2}}=\frac{F_{\mathrm{in}}Y_{{\mathrm{in}}_{{\mathrm{CO}}_{2}}}-F_{\mathrm{out}}Y_{{\mathrm{out}}_{{\mathrm{CO}}_{2}}}}{F_{\mathrm{in}}Y_{{\mathrm{in}}_{{\mathrm{CO}}_{2}}}}\times 100\%$$

3 $$r_{H_{2}\;\mathrm{or}\;\mathrm{CO}}=\frac{n_{H_{2}}{\mathrm{orn}}_{\mathrm{CO}}}{m_{\mathrm{cat}}}$$

4 $$r_{{\mathrm{CH}}_{4}\;\mathrm{or}\;{\mathrm{CO}}_{2}}=\frac{n_{{\mathrm{CH}}_{4\mathrm{in}}}-n_{{\mathrm{CH}}_{4\mathrm{out}}}}{m_{\mathrm{cat}}}$$

5 $$R_{H_{2}/\mathrm{CO}}=\frac{{\mathrm{rH}}_{2}}{r_{\mathrm{CO}}}$$

6 $$n=\frac{F\left(L{\cdot \min}^{-1}\right)\times \mathrm{vol}\%\;\left(\mathrm{mol}\;\mathrm{fraction}\right)}{22.4\left(L\cdot {\mathrm{mol}}^{-1}\right)}$$

where *n* is the molar flow rates of CH_4_, CO_2_, H_2_, and CO.

Using the Arrhenius equation, the logarithm of the carbon dioxide reaction rate and the reciprocal of temperature can be plotted to determine the activation energy from the slope using eq ([Disp-formula eq7]).

7 $$\ln_{{\mathrm{CO}}_{2}}=\frac{\mathrm{Ea}}{R}\left(-\frac{1}{T}\right)+\ln A$$

Here, *R* represents the molar gas constant (=8.314 J mol^–1^ K^–1^), *A* is the prefactor, and *T* is the temperature in *K*.

## Results and Discussion

### Catalyst Characterization

#### XRD Analysis

The XRD data for the pristine La_2_Ti_2_O_7_ and La_2_Ti_2–*x*_Ru_*x*_O_7_ catalysts with varying Ru contents are shown in Figure 2(a). All diffraction peaks can be indexed to the monoclinic layered perovskite structure of the La_2_Ti_2_O_7_ phase (space group *P*2_1_) which is in accordance with the standard diffraction data (JCPDS/PDF No. 70-0903, 81-1066, and ICDD 81-1066).^22,23^ The characteristic reflections located at 21.1° (012), 23.2° (002), 27.8° (013), 29.8° (210), 32.2°(014), 40.0°(220), 46.5° (401), 48.0° (215), 52.0° (413), and 57.8° (233) are attributed to the layered TiO_6_-octahedral framework of La_2_Ti_2_O_7_. These reflections are in good agreement with the characteristic planes of the layered perovskite La_2_Ti_2_O_7_ and show that the parent layered structure was successfully formed. The diffraction patterns of all samples do not change when the Ru content is increased, and no other crystalline phases appear, suggesting a structurally stable layered perovskite framework even after the addition of Ru. Also, no separate diffraction peaks of metallic Ru or rutile RuO_2_ can be seen, which indicates that the Ru species are embedded in the oxide lattice. The small increase in peak intensity and broadening of the peaks with increasing Ru content show local lattice distortion due to the incorporation of Ru into the lattice and the defects generated, which still maintain the same layered perovskite structure. The La_2_Ti_2_O_7_ framework is preserved after Ru addition, which is advantageous due to the presence of many interfacial sites, good charge transport, and high mobility of oxygen in the structure.

**Figure 2:**
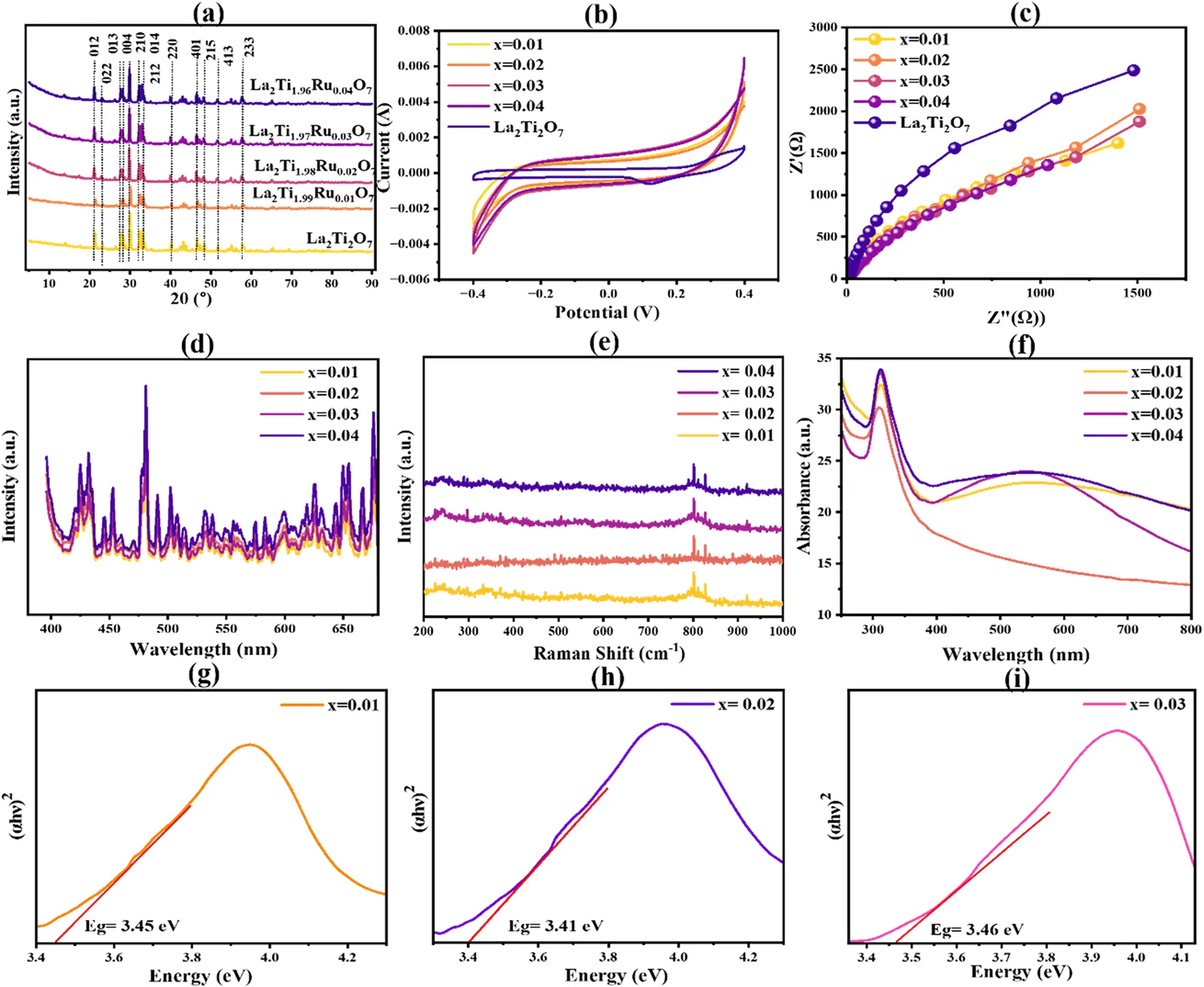
XRD, CV, EIS, PL, Raman, UV-DRS, Tauc plots. (a) XRD patterns of LTO, *x* = 0.01–0.04, (b) CV comparison of *x* = 0.01–0.04, (c) Nyquist EIS plots of *x* = 0.01–0.04, (d) PL of *x* = 0.01–0.04, (e) Raman spectra of *x* = 0.01–0.04, (f) UV-DRS of *x* = 0.01–0.04, and Tauc plot: (g) *x* = 0.01, (h) *x* = 0.02, (i) *x* = 0.03.

There are slight changes in the peak intensity and peak broadening with increasing Ru content, which can be seen from a careful comparison of the diffraction patterns and results from lattice distortion due to Ru incorporation. This structural change is due to the replacement of Ti by Ru ions, which have different ionic radii and electronic configurations and thus affect the local crystal environment. The preservation of the perovskite framework after the addition of Ru confirms that the parent crystal structure is structurally stable and that the addition of Ru does not cause any disruption of the structure. Moreover, the peak shift is not very prominent, indicating that only a small amount of Ru is incorporated into the lattice, as found in the low-level substitution (*x* = 0.01–0.04).

#### Cyclic Voltammetry (CV) Analysis

The electrochemical performance of La_2_Ti_2–*x*_Ru_*x*_O_7_ perovskites is studied by cyclic voltammetry (CV) within a potential range of −0.4 V to +0.4 V, with a scan rate of 100 mV s^–1^ to understand the effect of Ru incorporation on the redox behavior and charge-transfer ability of the catalysts. All the samples have quasi-rectangular CV profiles, as shown in Figure 2(b).

The CV profiles show a significant enhancement in the electrochemical response of layered La_2_Ti_2_O_7,_ with all compositions of La_2_Ti_2–*x*_Ru_*x*_O_7_ having a significantly higher current density and enclosed CV area. The insulating nature of pristine La_2_Ti_2_O_7_ is in line with the comparatively slow charge-transfer properties reported for this material, while changes in the titanate lattice structure can significantly affect the electrochemical properties of the material.^24^ The overall current response is higher for *x* = 0.03 and *x* = 0.04, and the anodic current is highest near the positive potential limit for *x* = 0.04. This shows that an intermediate-to-high Ru concentration is most beneficial for providing an electrochemically accessible electronic environment. The enlarged CV area upon Ru incorporation suggests an increase in charge accommodation and more facile electron transfer processes at the interface. Notably, the curves show a lack of strong, well-resolved redox peaks, indicating that the electrochemical behavior is associated with processes that are distributed across the surface or near the surface charge-transfer processes. This is in line with earlier investigations that have demonstrated that the electronic structure and electrochemical kinetics of layered La_2_Ti_2_O_7_ can be significantly changed by defect engineering. Mariappan et al. showed that for La_1.7_Ti_2_O_7_–_δ_, the CV current response was significantly higher than that of La_2_Ti_2_O_7_, and this was linked to the improvement in its electronic conductivity, number of accessible sites, and charge-transfer kinetics.^25^

#### Electrochemical Impedance Spectroscopy (EIS)

Electrochemical Impedance Spectroscopy (EIS) is used to explore the effect of Ru incorporation on charge-transfer properties and electrical conductivity of La_2_Ti_2–*x*_Ru_*x*_O_7_ perovskites, as shown in Nyquist plots in Figure 2(c). Pristine La_2_Ti_2_O_7_ shows a significantly large *Z*′ and *Z*″ response, and all La_2_Ti_2–*x*_Ru_*x*_O_7_ samples exhibit compressed impedance profiles, which suggests that charge transport is facilitated by the incorporation of Ru. For a conventional Nyquist response, the high-frequency intercept corresponds to the value of the series or solution resistance R_s_, and the high- to intermediate-frequency arc corresponds to the value of the interfacial charge-transfer resistance *R*_ct_. The smaller the arc or the lower the impedance response, the faster the interfacial electron-transfer kinetics.^26^ The impedance response curves for the Ru-substituted compositions indicate a comparatively low impedance response for *x* = 0.03 and *x* = 0.04, which indicates that these Ru concentrations are found to provide a good electronic environment for charge transport. This improvement can be attributed, as a reasonable possibility, to the Ru–Ti–O framework and the oxygen-defect environment, which can provide electronically accessible states and promote charge migration through the oxide lattice.

La_2_Ti_2–*x*_Ru_*x*_O_7_ has been shown to have a defect-mediated effect, wherein the electrochemical response is altered with increasing defect concentration, but for high levels of substitution. This has been shown to lead to lattice distortion and degradation of the electrochemical response. Additional low-frequency inclined features are due to diffusion/transport processes, which are often modeled as a Warburg element in equivalent-circuit analysis.^27^ In addition to the larger CV responses of the Ru-substituted samples, the lower EIS impedance confirms that the introduction of Ru improves the electrochemical charge-transfer properties of La_2_Ti_2_O_7_.

#### Photoluminescence (PL) Analysis

To study the recombination behavior of the generated charge carriers and the effect of substitution of Ru on the electronic structure of the perovskite, the photoluminescence (PL) spectra of La_2_Ti_2–*x*_Ru_*x*_O_7_ are presented in Figure 2(d).

All compositions of La_2_Ti_2–*x*_Ru_*x*_O_7_, on the other hand, exhibit a significant reduction in the PL broad background, suggesting that there are also other nonradiative/charge-transfer pathways that are created by Ru substitution and compete with radiative electron–hole recombination. The electronic states of the TiO_6_ polyhedral network can be significantly changed by perturbing the La_2_Ti_2_O_7_ lattice, and this can lead to changes in the process of excitation/energy transfer.^28^ The replacement of Ru atoms at Ti sites is anticipated to affect the local Ru–O–Ti coordination environment and introduce extra electronic levels that can accept or trap excited carriers. The integrated PL intensity of La_2_Ti_2–*x*_Ru_*x*_O_7_ is therefore considerably lower, which indicates that radiative carrier recombination was suppressed, but this does not necessarily indicate better charge separation because nonradiative defect recombination can also lead to PL quenching. The quenched background is overlaid with a few relatively sharp features in the vicinity of 420–455, 480, 490–510, and 530–590 nm. This suggests that Ru incorporation affects discrete radiative pathways associated with defects or lattices but does not simply reduce the intensity of the emission. The strong peak near 480 nm in the Ru-substituted materials could be the result of a change in the electronic environment of Ti–O or Ru–O. This demonstrates that the energy migration properties of La_2_Ti_2_O_7_ are highly sensitive to changes in the TiO_6_ connectivity and electronic state density, highlighting the effects of polyhedral perturbation on optical properties. The PL response, therefore, complements the CV and EIS observations, providing evidence that the substitution of Ru significantly enhances interfacial charge transfer.

#### Raman Spectroscopy

The lattice dynamics and structural evolution of La_2_Ti_2–*x*_Ru_*x*_O_7_ as a function of Ru substitution were investigated using Raman spectroscopy. The Raman spectra of all the compositions are presented in Figure 2(e), showing that the perovskite framework remained unchanged. The Ti–O framework vibration is the dominant feature around 800 cm^–1^, and weaker modes at lower wavenumbers can be attributed to this vibration, mostly Ti–O stretching in the distorted TiO_6_ units. No obvious new bands of high Raman intensities are seen, which indicates that there is no easily detectable Raman-active crystalline secondary Ru-rich phase within the measurement sensitivity. The changes in the intensity, broadening, and local peak structure in the 780–830 cm^–1^ region suggest that the coordination environment of Ti is perturbed upon the incorporation of Ru. These changes are like those expected because of lattice distortion and changes in Ru–O bonding in the La_2_Ti_2_O_7_ framework. The relatively wide spectral background also indicates some significant structural disorder and defect heterogeneity for these compositions.

#### UV–Vis (UV-DRS) Spectroscopy

The optical absorption properties of La_2_Ti_2–*x*_Ru_*x*_O_7_ perovskites were investigated by UV–vis diffuse reflectance spectroscopy (UV-DRS) to evaluate the influence of Ru substitution on their electronic structure and light-harvesting capability. As shown in Figure 2(f), all samples exhibit strong absorption in the ultraviolet region, corresponding to the intrinsic O 2p and Ti 3d charge-transfer transition of the titanate perovskite. Upon Ru incorporation, the absorption edge progressively extends into the visible region, indicating that Ru effectively modifies the electronic band structure and enhances visible-light utilization. The enhanced absorption is attributed to the introduction of Ru electronic states, which interact with Ti 3d and O 2p orbitals.^29^ The improved visible-light response is further associated with enhanced orbital hybridization and oxygen-vacancy-related defect states, which facilitate photon absorption and carrier excitation.^30^ These electronic modifications are expected to improve the generation of photoexcited charge carriers and strengthen the photothermal response under solar irradiation.

Tauc plots obtained from UV–vis diffuse reflectance data using the Kubelka–Munk transformation were used to estimate the band gap energies. The band gap energies are 3.45, 3.41, 3.46, and 3.48 eV for *x* = 0.01, 0.02, 0.03, and 0.04, respectively, as depicted in Figures 2(g*–*i) and 3(a). The optical band gaps of La_2_Ti_2_O_7_ remain almost unchanged (3.41–3.46 eV) with Ru incorporation, and the absorption extends into the visible region. The increased visible absorption is attributed to Ru/defect-associated sub-band gap states and changes in the local Ru–O–Ti electronic environment. The transition to a lower-energy state in such states does not significantly change the basic absorption edge. The minor *E*_g_ variations are possibly due to lattice distortions and/or perturbations in the Ti–O electronic structure caused by oxygen defects. Therefore, the enhanced light-harvesting and photothermal DRM performance of La_2_Ti_2–x_Ru_*x*_O_7_ is due to sub-band gap absorption, as opposed to band gap narrowing.

**Figure 3:**
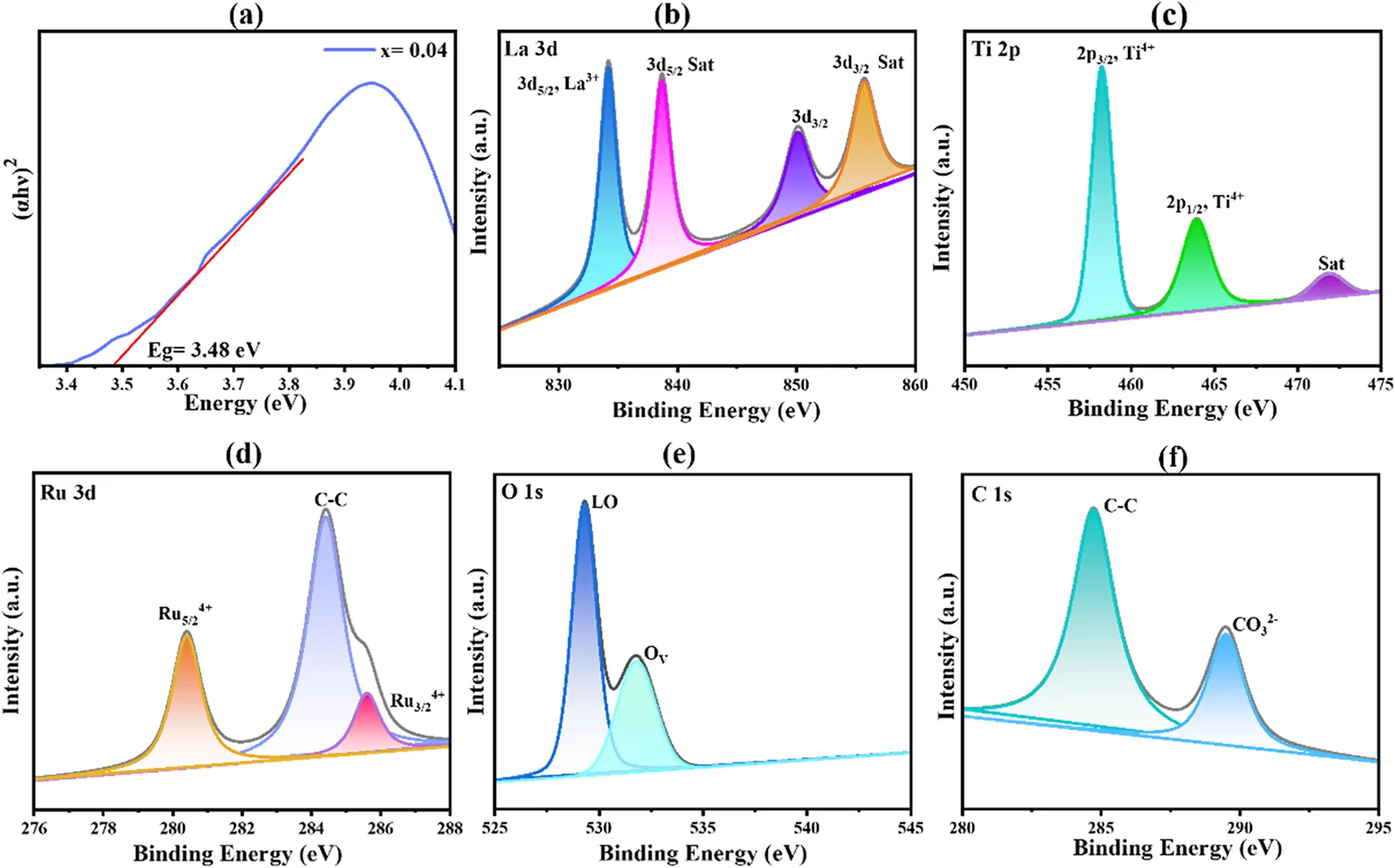
Tauc plot and XPS analysis of *x* = 0.04: (a) Tauc plot, (b) XPS spectra of La 3d, (c) Ti 2p, (d) Ru 3d, (e) O 1s, and (f) C 1s.

#### XPS Analysis

Direct evidence of electronic structure reconstruction and surface chemical evolution of the catalyst before and after the reaction is obtained from the high-resolution XPS spectra. The shifts in binding energies, changes in peak intensities, and changes in the oxidation states of the metals are all consistent with the presence of strong metal–support interactions and surface redox processes during catalytic activity. The La 3d spectrum in Figure 3(b) shows the 3d_5/2_ and 3d_3/2_ doublets characteristic of lanthanum in the trivalent state with prominent shakeup satellite peaks. These shakeup satellite peaks are characteristic of La^3+^ species which originate from final-state charge transfer. The Ti 2p spectra in Figure 3(c) show highly resolved peaks corresponding to the Ti^4+^ 2p_3/2_ (458 eV) and 2p_1/2_ (464 eV) states with characteristic satellite features. The lack of lower-energy Ti^3+^ components suggests that Ti occurs predominantly as Ti^4+^ under the conditions studied, and the shakeup satellite peak reinforces the typical electronic structure of a Ti–O bond in the oxide framework.

The Ru 3d XPS spectrum is shown in Figure 3(d). The Ru 3d_5/2_ peak at 280.5 eV is attributed to Ru^4+^, suggesting that Ru is mainly in an oxidized state in the La_2_Ti_2_O_7_ matrix. The strong peak at 284.8 eV is the adventitious carbon C 1s (C–C/C-H) peak, which also overlaps with the Ru 3d_3/2_ component.^31^ Thus, the Ru oxidation state was deduced from the Ru 3d_5/2_ region after proper deconvolution of the overlapping Ru 3d and C 1s signals.^32^ The O 1s spectrum presented in Figure 3(e) can be divided into lattice oxygen (O_L_, 529–530 eV) and oxygen vacancy (O_V_, 531–532 eV). The O_V_ shows higher oxygen vacancies, facilitating CO_2_ activation while promoting lattice oxygen mobility. O_L_ and O_V_ indicate a highly defective oxygen that enhances the dynamic lattice oxygen in photothermal DRM. The C 1s spectrum shown in Figure 3(f) has only a small peak of the C = C species and a small peak corresponding to the carbonate (CO_3_^2–^) species, but the C–C/C–H peak (284.8 eV) is dominant before the reaction. The small peak of carbonates indicates that the catalyst is resistant to carbon deposition. The presence of surface carbonates confirms CO_2_ adsorption on the catalyst surface and lattice oxygen mobility. XPS analysis showed that the catalyst experienced extensive electronic redistribution and enhanced metal–support interaction between Ru, Ti, and La-containing species.

#### SEM and TEM Analyses

The morphological and microstructural properties of the optimized La_2_Ti_2–*x*_Ru_*x*_O_7_ catalyst are shown in Figure S1. The SEM image in Figure 4(a) shows that the catalyst comprises agglomerated particles distributed evenly throughout the sample and has a relatively rough surface, which is desirable for reactant adsorption in the photothermal DRM process. Figure 4(b) illustrates that the particles appear as irregular nanosized grains, suggesting that a porous interconnected structure is formed, which may allow for mass transport and light harvesting.

**Figure 4:**
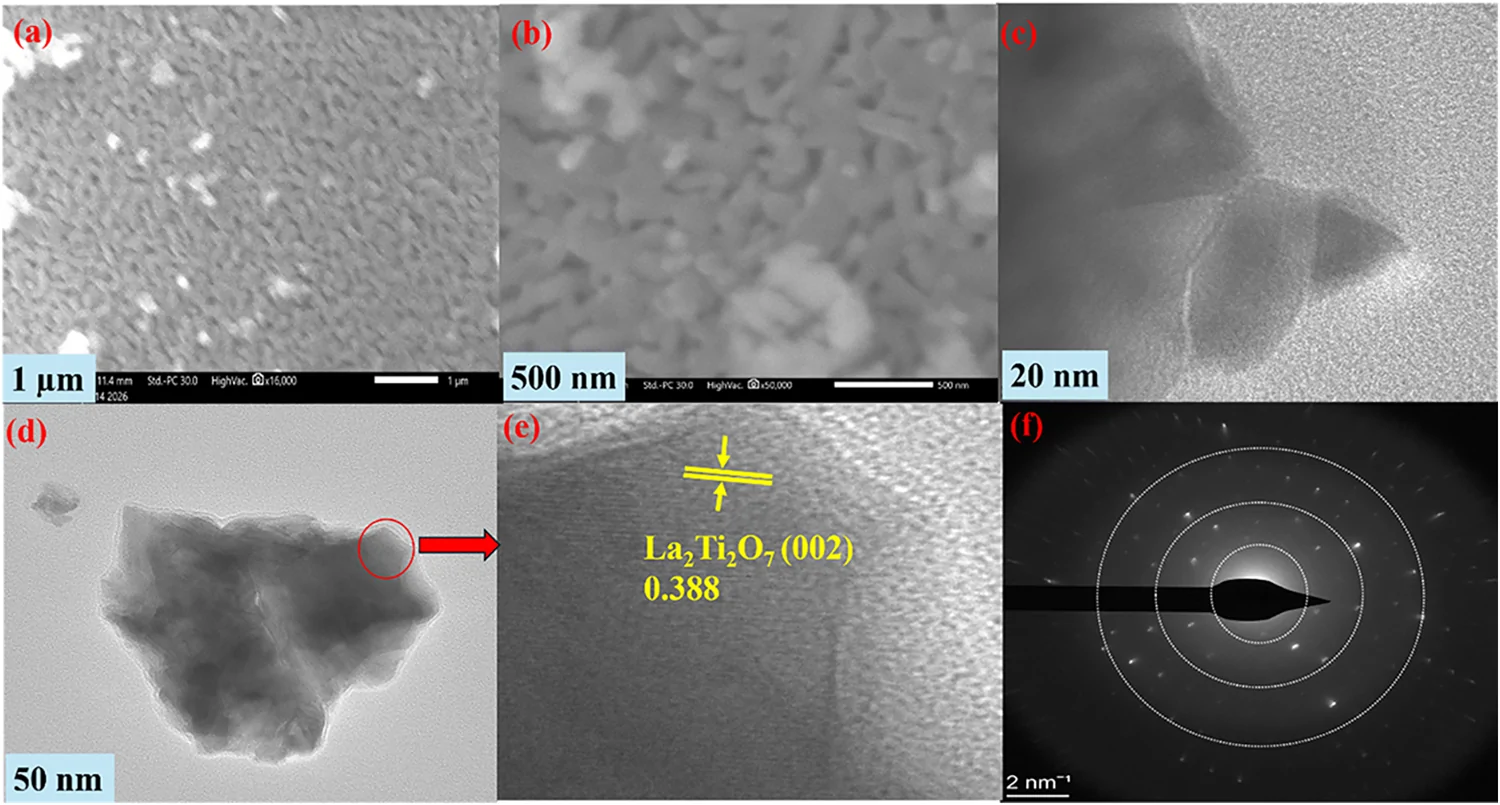
SEM, TEM, and EDS analyses of La_2_Ti_2_O_7_: SEM images: (a) *x* = 0.03 and (b) *x* = 0.04; TEM images (c, d), *d*-spacing of La_2_Ti_2_O_7_ (e), and SAED pattern of La_2_Ti_1.96_Ru_0.04_O_7_ (f).

Successful incorporation of Ru into the perovskite matrix was confirmed by the TEM image in Figure 4(c), which shows no noticeable phase segregation and large Ru nanoparticles without the presence of Ru nanocrystallites. The TEM image is shown in Figure 4(d), with the highlighted regions showing uniformly distributed crystalline domains. The TEM image in Figure 4(e) shows La_2_Ti_2–*x*_Ru_*x*_O_7_ with well-resolved and continuous lattice fringes, which confirms the crystalline nature of the layered perovskite structure. The interplanar spacing measured is about 0.388 nm, corresponding to the (002) crystallographic plane of the lattice of the type La_2_Ti_2_O_7_.^33^ The lattice fringes are sustained in order, suggesting that the crystalline framework is retained after Ru incorporation. The SAED pattern in Figure 4(f) shows clear diffraction spots, which corroborate the high crystallinity of the area.^22,23^ The close contact between these lattice planes suggests that the Ru–O–Ti interface structure is strong, which is likely to result in efficient charge transfer and fast oxygen migration in catalysis. The HAADF-STEM image in Figure S1 and the associated EDS elemental mapping images show that the concentration of all elements is uniform across the area of the catalyst. The successful incorporation of Ru into La_2_Ti_2–*x*_Ru_*x*_O_7_ (Figure S2) is evidenced by the uniform dispersion of elements, which also generates many Ru–O–Ti interfacial sites.

#### Photothermal Catalytic Performance

The catalytic behaviors of the catalysts La_2_Ti_2_O_7_, La_2_Ti_1.99_Ru_0.01_O_7_, La_2_Ti_1.98_Ru_0.02_O_7_, La_2_Ti_1.97_Ru_0.03_O_7_, and La_2_Ti_1.96_Ru_0.04_O_7_ were monitored under thermal and photothermal DRM conditions. The wavelength-dependent DRM results show that the yield of H_2_/CO decreases gradually from 267.85/384.12 mmol g^–1^ h^–1^ at 350 nm to 233.23/294.64 mmol g^–1^ h^–1^ at 600 nm, suggesting that the DRM process has a strong wavelength dependence (Figure S3). Even though the band gap is 3.48 eV, a significant activity indicates a contribution from the Ru-induced sub-band gap/interfacial states, as well as from photothermal conversion. Under visible-light irradiation, La_2_Ti_1.96_Ru_0.04_O_7_ achieves a temperature of 67 °C, while pristine La_2_Ti_2_O_7_ attains only 36 °C (Figure S4). This difference in temperature directly shows that the incorporation of Ru greatly improves the light-to-heat conversion efficiency. Taken together, these observations suggest that the increased DRM activity is due to the interaction between wavelength-dependent electronic excitation/interfacial processes and Ru enhanced by photothermal heating.

Pure La_2_Ti_2_O_7_ showed low H_2_ and CO rates under both thermal (4.7 and 5.3 mmol g^–1^ h^–1^) and photothermal (9.3 and 12.4 mmol g^–1^ h^–1^) conditions. La_2_Ti_2–*x*_Ru_*x*_O_7_, compared with bare La_2_Ti_2_O_7_, generated additional B-site active centers and thus improved the electronic structure of the perovskite lattice. The addition of Ru at *x* = 0.01–0.04 caused a marked increase in both H_2_ and CO production under thermal conditions, as shown in Figure 5(a). This confirms that Ru is effective in promoting the C–H bond cleavage and CO_2_ activation of the adsorbed species. In addition, the production of both H_2_ and CO is further promoted by the localized photothermal operation (Figure 5(b). Photothermal irradiation shows an enhanced effect on H_2_ and CO production compared with conventional thermal catalysis at the same reaction temperature. All Ru-substituted catalysts showed a synergistic effect between light and heat for methane reforming. Ru simultaneously acts as the major CH_4_ activation site and electron coupling site with the lattice of La_2_Ti_2–*x*_Ru_*x*_O_7_ at the Ru–O–Ti interface. This interfacial interaction enables fast electron transfer, reduces the activation energy for the cleavage of the C–H bond, and increases the generation of oxygen vacancies, which enhances the activation of CO_2_. Thus, Ru not only boosts the intrinsic catalytic activity but also improves the efficiency of the absorbed photon energy used in chemical reactions.

**Figure 5:**
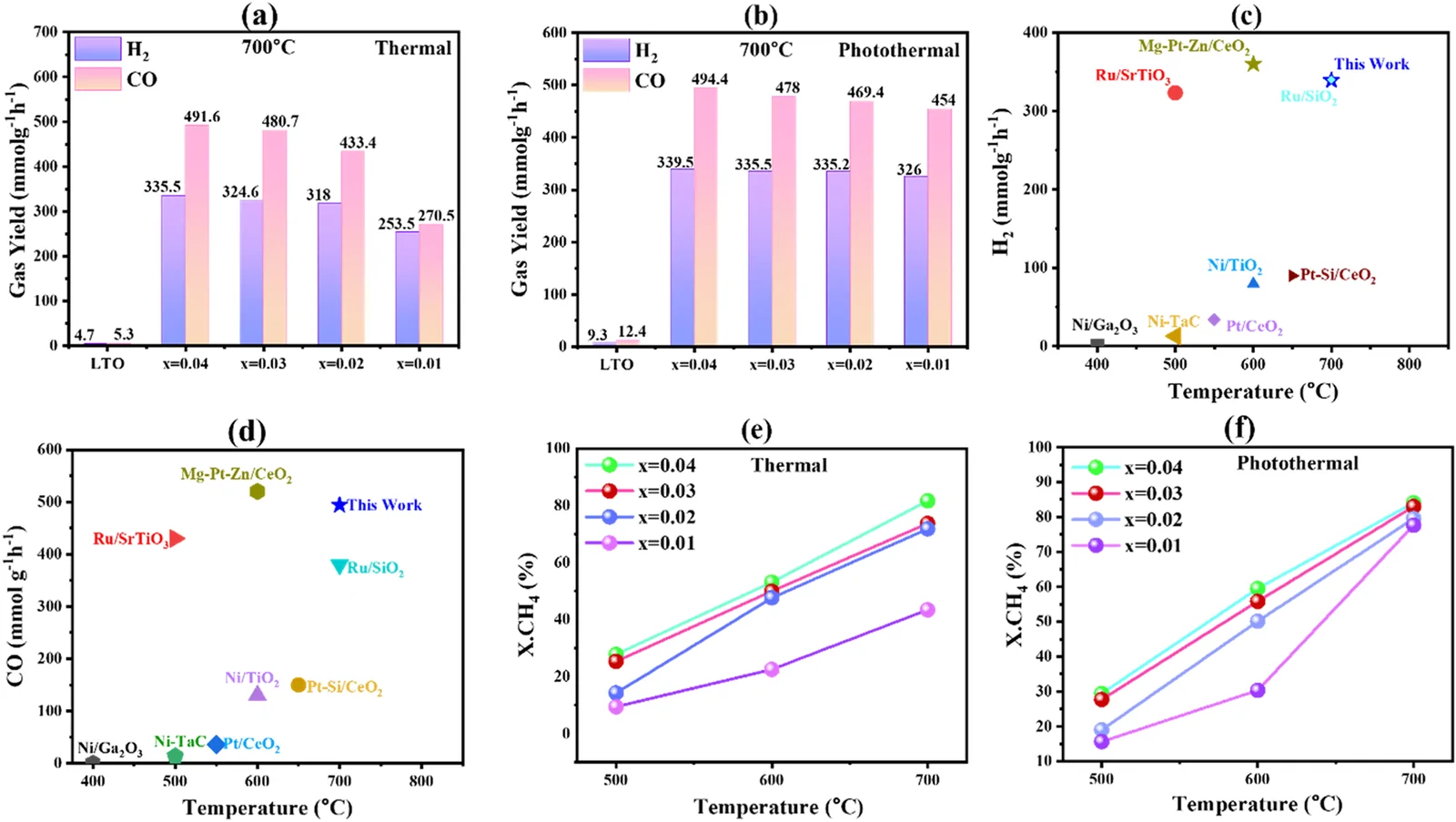
H_2_ and CO yields under (a) thermal and (b) photothermal conditions; CH_4_ conversion under (c) thermal and (d) photothermal conditions; CO_2_ conversion under (e) thermal and (f) photothermal conditions.

The photothermal DRM performance can be directly compared with the thermal DRM performance, which shows that illumination makes a definite contribution to the DRM performance, which is dependent on the temperature and composition. The H_2_ and CO production under photothermal conditions are close to those under thermal conditions at 700 °C, especially for x = 0.03 and 0.04, as shown in Figure 5(a,b). This suggests that the thermal activation process is dominant at high temperatures. In contrast, a more pronounced light-assisted enhancement is observed at 600 °C (Figures S5 and S7), where the H_2_/CO yields of *x* = 0.04 increase from 264.02/320 to 293.0/353.32 mmol g^–1^ h^–1^ and those of *x* = 0.03 increase from approximately 261/320 to 277/335 mmol g^–1^ h^–1^. The low thermal yields are further evidence of the high-temperature dependency of DRM at 500 °C (Figures S6 and S8). Based on these observations, illumination is taken as a photothermal contribution, which is most clearly seen at intermediate temperatures, where it adds to conventional thermal activation. Because of the dominant thermal pathway, the additional contribution of light is small at 700 °C. Yang et al.^13^ reported that H_2_ and CO yields were 450 and 520 mmol g^–1^ h^–1^ by using Ru and Au supported on SrTiO_3_ at 550 °C. Li et al.^34^ observed that H_2_ and CO yields were 320 and 420 mmol g^–1^ h^–1^ by using Ru supported on TiO_2_. Similarly, Kang et al.^35^ claimed the yields of H_2_ and CO to be 632 and 526 mol g_Ru_^–1^ h^–1^ using Ru on multiwalled carbon nanotubes at 600 °C. Rao et al. reported lower rates of H_2_ and CO (2 × 10^–4^ and 2.2 × 10^–4^ molg^–1^ h^–1^) at 400 °C by using Ni/Ga_2_O_3_. A comparison of H_2_ and CO rates under photothermal conditions from the literature, based on catalysts Ni-TaC,^36^ Pt/CeO_2_,^37^ Ni/TiO_2_,^38^ Pt–Si/CeO_2_,^39^ Ru/SrTiO_3_,^40^ Ru/SiO_2_,^41^ and Mg–Pt–Zn/CeO_2_ ^42^ is shown in Figure 5(c,d). The comparison illustrates that La_2_Ti_2–*x*_Ru_*x*_O_7_ has better rates of production of H_2_ and CO from different catalysts in photothermal conditions. The conversion profiles of CH_4_ in Figure 5(e,f) both show a steady rise as the reaction temperature increases for both thermal and photothermal reactions. La_2_Ti_1.96_Ru_0.04_O_7_ showed CH_4_ conversions of 27.8%, 53.09%, and 81.66% at 500 °C, 600, and 700 °C, respectively, under thermal conditions. This increased by 29.37%, 59.47%, and 84.08%, respectively, under photothermal conditions.

Similarly, CO_2_ conversions increased from 28.9%, 54.5%, and 83.93% to 34.63%, 67.95%, and 85.92% in thermal and photothermal conditions Figure 6(a,b). Zhang et al.^43^ observed that the consumption rates of CH_4_ and CO_2_ increased under light irradiation on a Pt/TiO_2_ catalyst. Su et al.^21^ observed CH_4_ and CO_2_ conversions of 40% and 50%, respectively, under photothermal conditions on Ru/BaTiO_3_. Wang et al.^44^ reported >90% conversion of CH_4_ and CO_2_ using the Ru on CeO_2_ at 700 °C. Li et al.^12^ studied the light-induced electronic effect of Ru on LaFeO_3_ and reported 38% more CH_4_ conversion under light irradiation. In another study, they observed photothermal DRM on LaMnFeO_3_ and reported 50% conversion at 500 °C.^11^ Yao et al.^45^ studied Rh/LaNiO_3_ and reported very high conversions of 90–95% for CH_4_ and CO_2_ at 550 °C. This is due to the positive temperature dependence of the dissociation of CH_4_, activation of CO_2_, and movement of reaction intermediates on the surface.^20^ The advantage of the photon-induced processes over the conventional thermal catalysis is evident from the consistently high CH_4_ and CO_2_ conversions obtained at the same temperature. The energetic charge carriers, along with the creation of localized hot spots, enable electron transfer to adsorbed CO_2_ molecules and at the same time weaken the first C–H bond of the methane molecule.^46^ The increased enhancement at intermediate temperatures implies a cooperative effect between thermal activation and electronic excitation induced by light irradiation. However, the smaller difference at higher temperatures implies that the thermal activation effect is more dominant.

**Figure 6:**
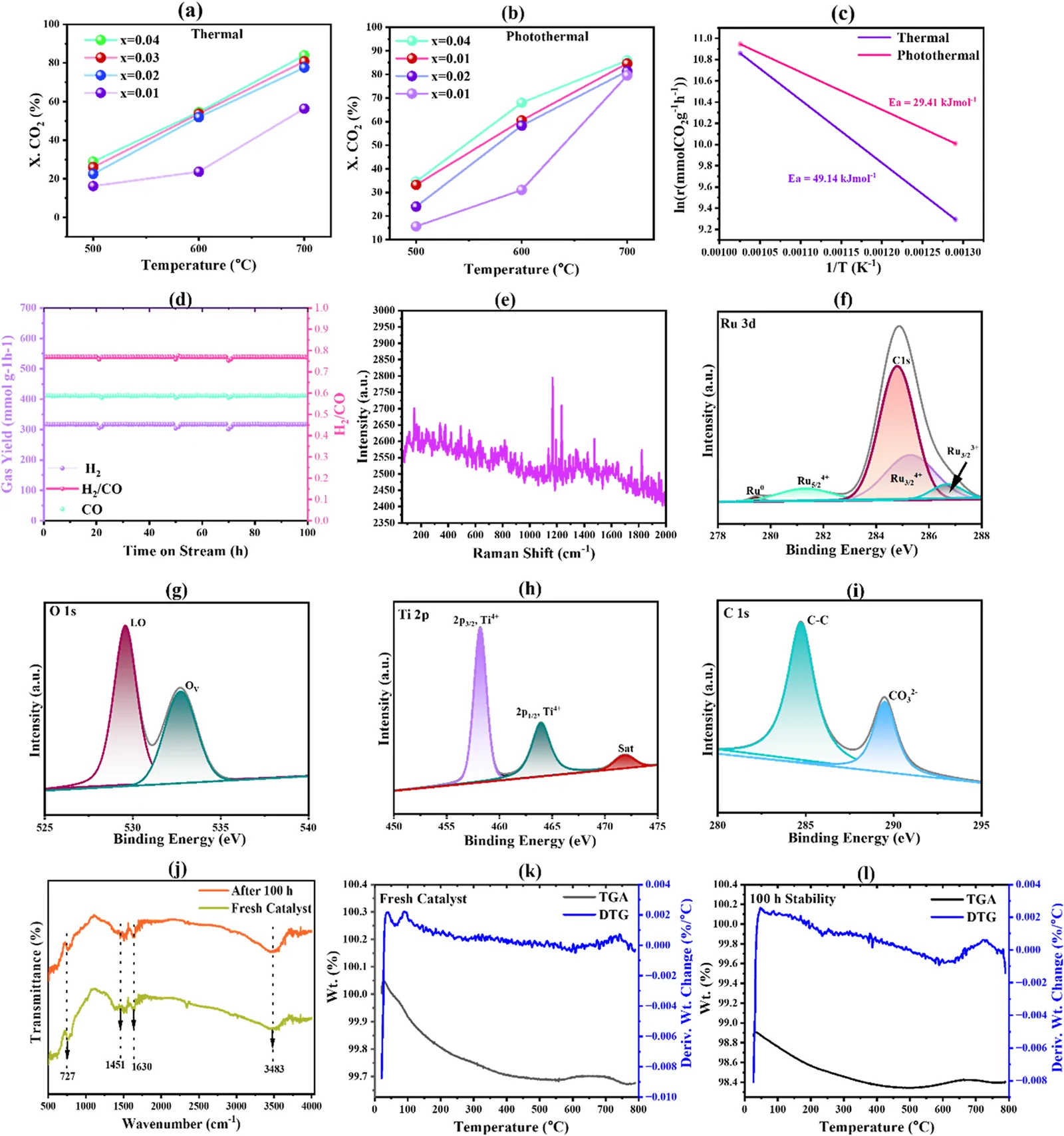
(a) Comparison of H_2_ and (b) CO gas yields. (c) Activation energy plot from the Arrhenius equation, (d) stability hours, gas yields of H_2_ and CO, and H_2_/CO ratio, (e) Raman spectra of the spent catalyst, XPS of the spent catalyst (*x* = 0.04) of (f) Ru 3d, (g) O 1s, (h) Ti 2p, and (i) C 1s. (j) FTIR analysis of fresh and spent catalysts, and TGA and DTG analysis of (k) fresh and (l) spent catalysts.

The H_2_/CO ratio is observed to be less than unity for all of the Ru-substituted catalysts. This suggests that DRM proceeds with a slight reverse water–gas shift (RWGS) reaction to remove a portion of the H_2_ produced to generate CO. The Arrhenius plots presented in Figure 6(c) confirm that the apparent activation energy in photothermal DRM is Ea = 29.41 kJ mol^–1^. This is much lower compared to the thermal operation (Ea = 49.14 kJ mol^–1^), which clearly establishes the direct kinetic evidence that light irradiation lowers the activation energy for the methane reforming process. The lower activation energy indicates that the photoexcited carriers are involved in the surface bond activation process and not just increasing the temperature of the catalyst.^45^

The long-term stability test shown in Figure 6(d) illustrates that the production rates of H_2_ and CO are maintained during the thermal reaction period of 100 h, with a H_2_/CO ratio in the range of 0.7, proving excellent structural stability and resistance to catalyst deactivation. Tang et al.^40^ reported 30 h of operation without a decrease in syngas yields on Ru/SrTiO_3_. Yang et al.^13^ claimed 40 h stability by using Ru–Au supported on perovskite SrTiO_3_ in a pressurized reactor of 0.1 MPa. Wang et al.^44^ claimed 100 h of maintained stability time on Ru/CeO_2_ nanorods at 700 °C. Duan et al.^20^ showed a higher stability time on Ru/BaTiO_3_ of 120 h which shows its high durability in photothermal conditions. Figure 6(e) shows no significant peaks of the D-band (1340–1360 cm^–1^) and G-band (1580–1600 cm^–1^), indicating that amorphous and graphitic carbon is much less, and hence negligible coke deposition.

The XPS of the spent catalyst further indicates that the photothermal DRM process does not cause phase decomposition of the perovskite structure. There is a strong Ru–O–Ti interfacial interaction, as evidenced by the presence of both Ru species and the La_2_Ti_1.96_Ru_0.04_O_7_ lattice, as shown in Figure 6(f), which can supply both complementary active sites for CH_4_ activation and CO_2_ conversion. The strong O_V_ feature in the O 1s spectrum in Figure 6(g) indicates that there are many defect features at the surface after the reaction. Similarly, the La 3d spectrum in Figure (S9) shows that it retained the La^3+^ 3d_5/2_ and 3d_3/2_ states. For Ti 2p, there is no significant change observed in the spent analysis, as shown in Figure 6(h). The high-resolution C 1s spectrum in Figure 6(i) shows two peaks: one centered at 284.8 eV, attributed to adventitious C–C and C–H species; and another peak at 289.2 eV, attributed to surface carbonate (CO_3_^2–^) species. The presence of carbonate species indicates successful CO_2_ adsorption and activation on the catalyst surface, whereas the lack of other high-binding-energy carbon species indicates the minimal accumulation of stable carbonaceous deposits. The XPS survey of the fresh and spent catalysts in Figure (S10) shows that the surface species La, Ti, O, and C are in the principal surface composition before and after the reaction.

The FTIR spectra of both fresh and 100 h spent La_2_Ti_1.96_Ru_0.04_O_7_ catalysts show almost identical vibrational features, which also indicates the excellent structural stability of the Ru-engineered perovskite structure during prolonged photothermal DRM. The broad absorption at 3483 cm^–1^ and the weak feature at 1630 cm^–1^ correspond to the stretching and bending modes of surface OH groups and physiosorbed water, respectively, as depicted in Figure 6(j). This suggests that the surface OH species are not substantially affected by the reaction. The presence of weak bands in the 1451 cm^–1^ range is attributed to the surface carbonate/bicarbonate species resulting from the adsorption of CO_2_ on the catalyst surface, which further supports the presence of CO_2_-derived intermediates on the catalyst surface. The characteristic lattice vibrations of Ti–O, Ti–O–Ti, and Ru–O–Ti are found at a low wavenumber of 727 cm^–1^ and do not change significantly after a reaction time of 100 h. This indicates that Ru incorporation does not adversely affect the integrity of the perovskite framework during the reaction. Most importantly, no extra absorption bands are detected after 100 h of operation, indicating that there was no irreversible accumulation of carbon. Both the lattice vibrations and surface oxygen-containing species are preserved, which supports the high structural strength and catalytic activity of the catalyst.

The spent La_2_Ti_1.96_Ru_0.04_O_7_ catalyst is then characterized by SEM, which shows that the perovskite particles remain mostly unchanged in shape following 100 h of photothermal DRM, as presented in Figure S11­(a–d). The EDS spectrum acquired at the same time confirms that no impurity elements are detected and shows the excellent structural integrity as well as compositional stability of the Ru-engineered catalyst after long operation (Figure S11­(e)). TEM analysis of the spent catalyst shows faceted crystalline particles with good lattice order (Figure S12­(a–d)). The elements in the catalyst, even Ru, are retained, as shown in EDS (Figure S12­(e)). The SAED pattern shows high crystallinity and an ordered phase on sharp and well-distributed sites (Figure S12­(f)).

Both fresh and 100 h spent catalysts show less than 0.5% weight loss in the temperature range from 25 to 800 °C which indicates the excellent thermal stability of the Ru-engineered perovskite in Figure 6(k–l). The minor weight loss below 200 °C is due to the desorption of physically adsorbed moisture and surface hydroxyl species. Moreover, no obvious DTG peaks were observed at higher temperatures, which suggested that no structural decomposition or phase transformation occurred. More importantly, there is no obvious carbon combustion peak on the spent catalyst, indicating that there is no coke deposition after long-term photothermal DRM. These results confirm the excellent structural strength of the catalyst.

These results indicate that controlled Ru substitution is an effective tuning method for the electronic structure, oxygen mobility, and charge-transfer properties at the interfaces of La_2_Ti_2–*x*_Ru_*x*_O_7_, which facilitates efficient, stable, and energy-efficient photothermal DRM. The best catalytic performance achieved for La_2_Ti_1.96_Ru_0.04_O_7_ indicates the significance of the optimal ratio of active-site density to lattice stability for the efficient use of photothermal catalysis.

#### In Situ DRIFT Analysis

The in situ DRIFTS spectra obtained at various temperatures for thermal (dark) and photothermal (light) DRM conditions are compared in Figure 7(a–c). The intense CH_4_ stretching vibrations were observed at 3005–3015 cm^–1^, and the characteristic asymmetric stretching bands of CO_2_ were observed at 2335–2360 cm^–1^. The intensity of both CH_4_ and CO_2_ bands decreases with the gradual progress of the reaction temperature from 500 °C to 600 and 700 °C under thermal conditions, which signals the progressive consumption of the reactants during DRM, as illustrated in Figure S13. However, the photothermal irradiation is significantly higher, especially at 600 and 700 °C, where the reactant activation and consumption on the surface is greatly activated. The high attenuation rate of the gaseous CO_2_ bands indicates that localized photothermal heating effects lead to greater CO_2_ adsorption, activation, and conversion into other products, rather than just higher adsorption capacity.^47^ The combined depletion of both CH_4_ and CO_2_ is observed under illumination, which is also supported by the catalytic measurements, where higher conversions are observed under illumination. Thereby, it supports the synergistic effect of the thermal energy and photoinduced electronic excitation.

**Figure 7:**
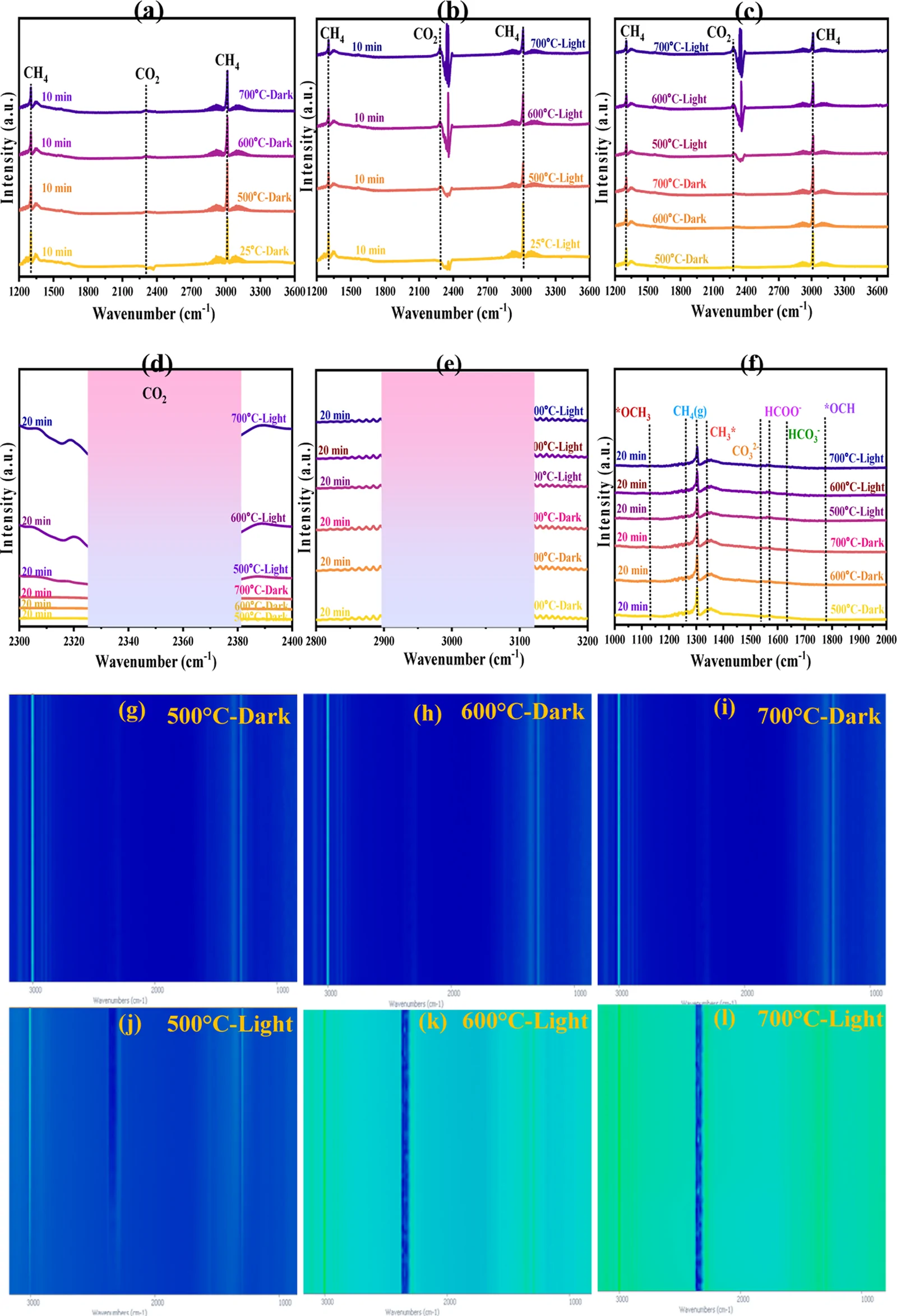
In situ DRIFT analysis: (a) thermal temperatures, (b) photothermal temperatures, (c) comparison of thermal and photothermal temperatures, (d) CO_2_ and (e) CH_4_ profiles, and (f) comparison of intermediates in the range of 1000–2000 cm^–1^. Time-dependent in situ DRIFT spectra: (g) 500 °C thermal, (h) 600 °C thermal, (i) 700 °C thermal, (j) 500 °C light, (k) 600 °C light, and (l) 700 °C light.

Figure 7(d) follows and expands the CO_2_ stretching region (2300–2400 cm^–1^), to better illustrate the effect of the photothermal irradiation on the activation of CO_2_. At elevated temperatures, the gaseous CO_2_ bands are still relatively strong under thermal conditions, which indicates that the activation of CO_2_ is not very fast. Photothermal operation, on the other hand, shows a significant decrease in CO_2_ absorption intensity, particularly at 700 °C, suggesting fast transformation of the adsorbed CO_2_ to reactive carbonate and oxygen-containing intermediates.^48^ This indicates more adsorption at the surface because electron transfer is more efficient to the antibonding levels of CO_2_ molecules from the photoexcited catalyst. This behavior is typical of highly active photothermal catalysts, where the turnover of intermediates is higher than their accumulation.^49^ CO_2_ activation is enhanced, consistent with the increased CO_2_ conversion seen during catalytic testing. This is believed to be the result of a combination of localized photothermal heating, enhanced oxygen-vacancy activity, and improved electronic conductivity provided by Ru substitution.

Figure 7(e) shows the CH_4_ stretching region at 2800–3200 cm^–1^. The progressive decrease in CH_4_ intensity with increasing temperature, under both thermal and photothermal conditions, confirms the progressive consumption of CH_4_. Interestingly, the CH_4_ stretching band is still quite intense during photothermal irradiation, especially at 700 °C, suggesting a much higher methane activation rate. Furthermore, the reduction in CH_4_ surface coverage under illumination directly confirms that photoinduced hot carriers work together with localized heating to decrease the activation barrier for the dissociation of methane.^50^ The rapid consumption of CH_4_ species matches well with the increased rate of H_2_ production under photothermal conditions. This indicates that Ru substitution is an effective catalyst to facilitate electron transfer to the adsorbed CH_4_ species, leading to sequential dehydrogenation to CH_*x*_ species.

The changes in the surface reaction intermediates in the region of 1000–2000 cm^–1^ are shown in Figure 7(f). During DRM, multiple oxygen-containing intermediates are identified, such as OCH_3_ (1134 cm^–1^), CH_4_ (1268, 1300, and 1345 cm^–1^), CO_3_^2–^ (1538 cm^–1^), HCOO^–^ (1580 cm^–1^), HCO_3_^–^ (1645 cm^–1^), and *OCH (1783 cm^–1^). The time-dependent spectral images under thermal and photothermal conditions at 500 °C, 600, and 700 °C are shown in Figure 7(g–l), illustrating the comparison of intensities. In thermal catalysis, these intermediates build up slowly as the temperature increases, while photothermal irradiation results in a marked variation in the intensity and distribution of these intermediates with temperature.^51^ The bands at 1268, 1300, and 1345 cm^–1^ are mainly attributed to CH_4_ adsorption/vibrational modes, suggesting the interaction between methane and the surface of the catalyst. The illumination-enhanced emergence/intensification of the bands at OCH_3_ (1134 cm^–1^) and *OCH (1783 cm^–1^) is a signature of increased conversion of CH_4_-derived species to oxygenated species, like the light-promoted pathway for Rh/TiO*_x_*/TiC.^52^ At the same time, the bands at CO_3_^2–^ (1538 cm^–1^), HCOO^–^ (1580 cm^–1^), and HCO_3_^–^ (1645 cm^–1^) are related to CO_2_ adsorption/activation by carbonate/formate-type surface species. The improved evolution under illumination suggests that there is an activation of CO_2_ on the surfaces as well as activation of CH_4_.^53^ Therefore, the more favorable OCH_3_/OCH reaction under light is consistent with increased oxygen-assisted transformation of CH_4_-derived intermediates as compared with the dark reaction. The overall light/dark comparison indicates an oxygen-mediated reaction pathway, which is favored by light. This behavior demonstrates that photothermal excitation increases both methane dehydrogenation and CO_2_ activation, resulting in more rapid surface redox cycles and continuous replenishment of O_2_.

#### Reaction Mechanism

The in situ DRIFTS results show the formation and evolution of surface intermediates during the photothermal DRM reaction over La_2_Ti_2–*x*_Ru_*x*_O_7_, which is consistent with the lattice-oxygen-assisted Mars-van Krevelen (MvK)-type pathway (Figure 8). Illuminated La_2_Ti_2–*x*_Ru_*x*_O_7_ can produce a photothermal effect and charge carriers (eq 8), which can work at the Ru^δ+^ sites to facilitate the activation of CH_4_. The Ru–O deficient sites also facilitate the activation of CO_2_. The dissociative activation of CH_4_ produces CH*_x_* and H species (eq 9), while CO_2_ adsorbs at the oxygen-deficient sites to produce activated carbonate/carbonate species (eq 10). Li et al.^8,52^ proposed species interactions involve CH*_x_* * reacting with nearby catalytic lattice oxygen O_L_ to create oxygenated CH_3_O*/CHO* species which further evolve to adsorbed CO* (eq 11). The consumption of lattice oxygen during the oxidation of CH*_x_* creates oxygen-deficient sites Vo (eq 12), which are proposed to be responsible for the adsorption and dissociation of CO_2_ to yield CO and to replenish the lattice oxygen consumed in the previous step (eq 13). The H* species recombine to form H_2_, while CO* desorbs as CO, forming syngas.

8 $${\mathrm{La}}_{2}{\mathrm{Ti}}_{2}-{x\mathrm{Ru}}_{x}O_{7}+\mathrm{h\upsilon}\to e^{-}+h^{+}+\mathrm{heat}$$

9 $${\mathrm{CH}}_{4}+{\mathrm{Ru}}^{\delta +}\to {\mathrm{CH}}_{x}+H^{*}$$

10 $${\mathrm{CO}}_{2}+\mathrm{Vo}\to {\mathrm{CO}}_{2}^{*}$$

11 $${\mathrm{CH}}_{x}+O^{*}\to {\mathrm{CH}}_{3}O^{*}$$

12 $${\mathrm{CH}}_{x}^{*}{+O}_{L}\to {\mathrm{CH}}_{x}O+\mathrm{Vo}$$

13 $${\mathrm{CO}}_{2}+\mathrm{Vo}\to {\mathrm{CO}}^{*}+O_{L}$$

**Figure 8:**
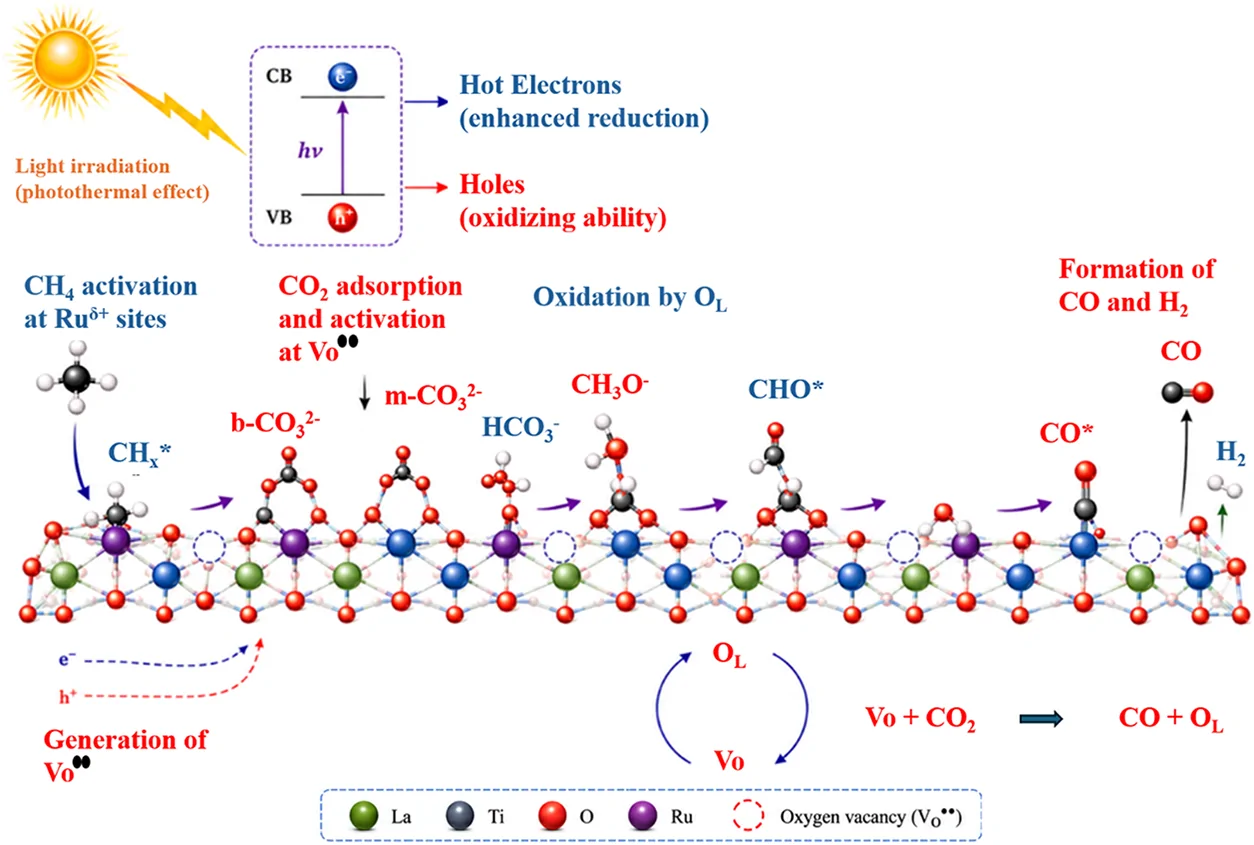
Illustration of the schematic reaction mechanism of oxygen-assisted Mars–van Krevelen mechanism for photothermal DRM on La_2_Ti_2–*x*_Ru_*x*_O_7_.

It provides sustained oxidation of CH_*x*_ fragments and rapid regeneration of active sites. Therefore, Ru is the main center of CH_4_ activation, while the Ti–O lattice is responsible for mobile oxygen species involved directly in the oxidation reaction. Therefore, the apparent activation barrier is reduced, oxygen transfer is faster, and the conversion of intermediates is increased. The surface deposition of carbonaceous species is reduced, and consequently, CH_4_ and CO_2_ conversions are higher, the rate of syngas production is higher, and the stability of the catalyst is higher than that of conventional thermal DRM.

## Conclusion

In this work, La_2_Ti_2–*x*_Ru_*x*_O_7_ was synthesized as an efficient, stable, and carbon-suppressing catalyst for photothermal DRM by both electronic structure and lattice oxygen engineering. Ru incorporation causes a significant enhancement in light harvesting, charge transfer, oxygen-vacancy formation, and Ru–O–Ti interfacial interactions, which results in high catalytic activity and stability. In situ DRIFTS and XPS studies showed that the reaction takes place by a lattice oxygen-supported Mars–van Krevelen mechanism. CH_4_ is activated on the Ru surface, lattice oxygen interacts with CH_*x*_ intermediates, and coupled cycles of lattice oxygen and Ru^δ+^ provide a source of oxygen vacancies. The combined effect of photothermal heating, generation of charge carriers, and dynamic lattice oxygen chemistry enhances the migration of oxygen and makes it possible to produce efficient and stable syngas for 100 h. The FTIR, SEM, TGA, and DTG results also support the suppression of carbon deposition to a greater extent. Overall, the results show that Ru incorporation is an effective method to achieve the tailoring of the lattice oxygen activity of perovskites, which can be used to develop advanced perovskite catalysts for photothermal DRM.

## Supplementary Material



## Notes

Experimental details, materials, equations, and in situ drift spectra, including photographs (PDF)
