# Supplementary material for: Ruδ+-Engineered La2Ti2–x Ru x O7 Perovskite with Dynamic Lattice Oxygen Vacancies for Efficient and Stable Photothermal Dry Reforming of Methane
Source: Langmuir. 2026 Oct 1;42(40):30413–27. doi: 10.1021/acs.langmuir.6c05014 (PMC13674220; doi:10.1021/acs.langmuir.6c05014)
Supplement: Supplementary file 1 [file la6c05014_si_001.pdf]

Supplementary Information

$Ru^{\delta+}$-engineered $La_2Ti_{2-x}Ru_xO_7$ Perovskite with Dynamic Lattice Oxygen Vacancies for Efficient and Stable Photothermal Dry Reforming of Methane

Kamran Kamran, Muhammad Tahir*

*Chemical and Petroleum Engineering Department, United Arab Emirates University,

P.O.BOX 15551, Al Ain, United Arab Emirates.

*Corresponding Author Email: muhammad.tahir@uaeu.ac.ae

Contents

1. **Text S1-S2** **S2**
2. **Equations S1-S6** **S3**
3. **FiguresS1-S13** **S4**

1. **Text S1-S2**

1. Materials & Characterization

Lanthanum Nitrate Hexahydrate La $(NO_3)_3.6H_2O$, Titanium tetra-iso-propoxide, (TTIP) (Ti $(OCH(CH_3)_2)_4$), Ruthenium Chloride Trihydrate $RuCl_3.3H_2O$, Ethylene Glycol $(CH_2OH)_2$ purchased from Sigma Aldrich and Citric acid ($C_6H_8O_7$) purchased from DUKSEN.

X-ray diffraction (XRD) analysis was done through a Rigaku Smart Lab diffractometer utilizing Cu-Kα radiation with 40 mA, 40 kV and 0.15 nm current, voltage and wavelength, respectively. The scanning speed was 0.02° min^{-1} and the range for 2θ was set at 3-100°. Fourier-transform infrared spectroscopy (FTIR) was performed on a Mattson/U. S. A. Genesis-II infrared spectrometer. The spectra (averaged 20 scans) were taken from ≤5 wt.% KBr supported discs of test oxides over the frequency range 4000-400 cm^{-1} at the resolution of 4 cm^{-1}. CV measurements are performed on electrochemical station CS-350 to get the plots between current density and voltage over voltage window from -1.5V to +1.5V. The X-ray photoelectron spectroscopy (XPS) was used on Axis ultra-DLD Shimadzu to determine the elemental states. The DRIFTS measurements were performed in a Nicolet™ iS50 FTIR spectrometer (Thermo-Scientific) at in-situ conditions in a Praying Mantis (Harrick) in high temperature reaction chamber. A separate thermocouple was used right below the catalyst bed to measure the temperature. The catalyst mass having 5mg, was mixed with 95mg of KBr in a fine powder to enhance the absorptivity.

Text S2:

Calculations for conversion, yields and ratio

The conversion of CO_2 and CH_4 (X_{CH4} and X_{CO2}), rate of H_2 and CO ($r_{H2 \text{ and } CO}$), and H_2/CO ratio ($R_{H2/CO}$) were calculated through the following equations:

2. Equations S1-S6

$$X_{CH_4}=\frac{F_{in}Y_{in_{CH_4}}-F_{out}Y_{out_{CH_4}}}{F_{in}Y_{in_{CH_4}}}\times 100\% \qquad \text{S1}$$

$$X_{CO_2}=\frac{F_{in}Y_{in_{CO_2}}-F_{out}Y_{out_{CO_2}}}{F_{in}Y_{in_{CO_2}}}\times 100\% \qquad S2$$

$$r_{H_2 \text{ or } CO}=\frac{n_{H_2} \text{ or } n_{CO}}{m_{cat}} \qquad S3$$

$$r_{CH_4 \text{ or } CO_2}=\frac{n_{CH_4in}-n_{CH_4out}}{m_{cat}} \qquad S4$$

$$R_{H_2/CO}=\frac{rH_2}{r_{co}} \qquad S5$$

$$\ln_{CO_2}=\frac{Ea}{R}\left(-\frac{1}{T}\right)+\ln A \qquad S6$$

where n is denoted by molar flow rates CH_4, CO_2, H_2 and CO. Using Arrhenius equation, the logarithm of carbon dioxide reaction rate and reciprocal of temperature can be plotted to find the activation energy from the slope. R represents the molar gas constant whose value is 8.314 $Jmol^{-1}K^{-1}$ and A is the pre-factor and T is temperature in K.

3. Figure S1-S13

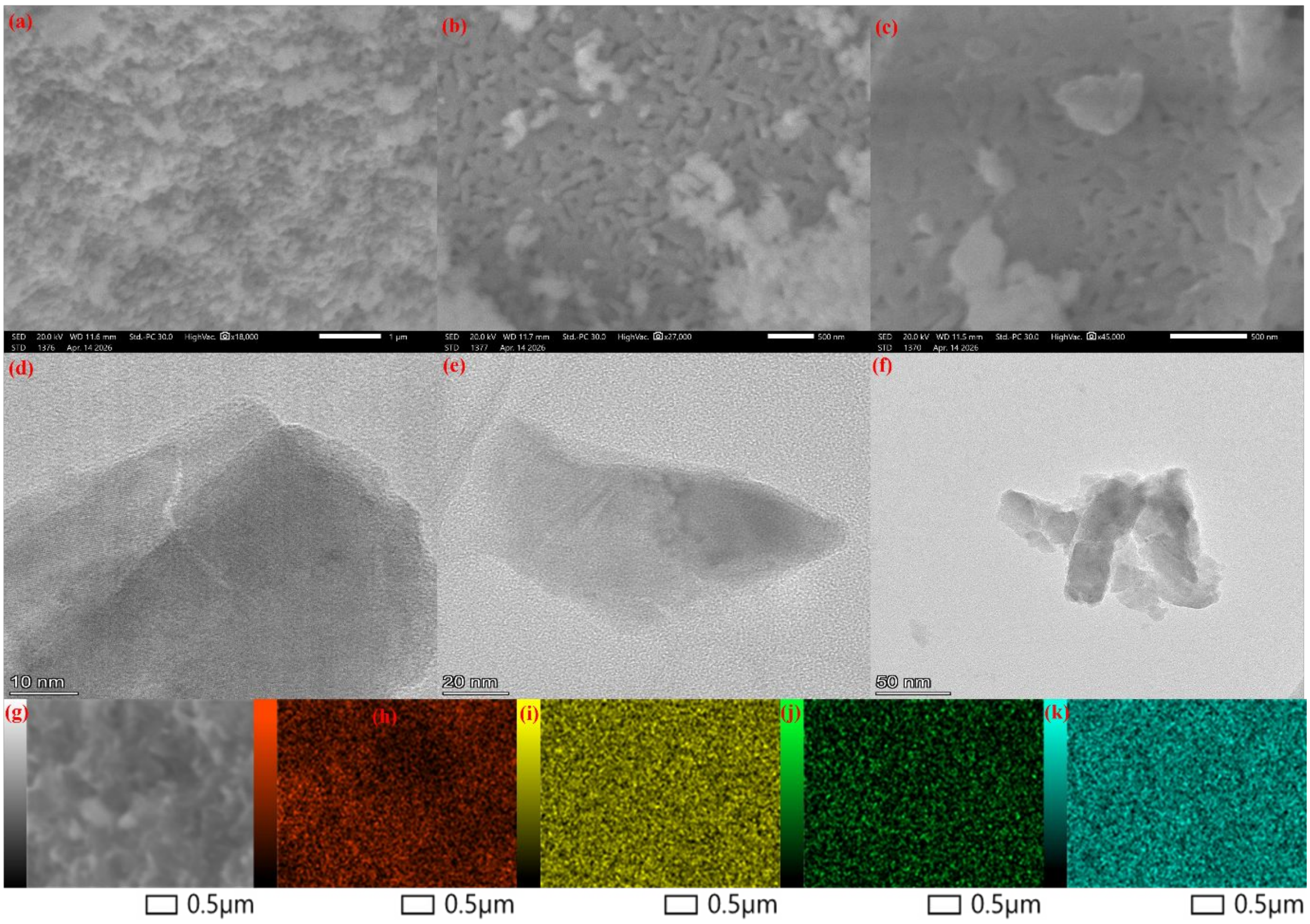

Figure S1: SEM images (a) x=0.01, (b) x=0.02, (c) x=0.04, TEM and EDS of x=0.04 (d-f), (g) HAADF-STEM and elemental mapping of x=0.04 (h) La, (i) Ru, (k) Ti, (k) O.

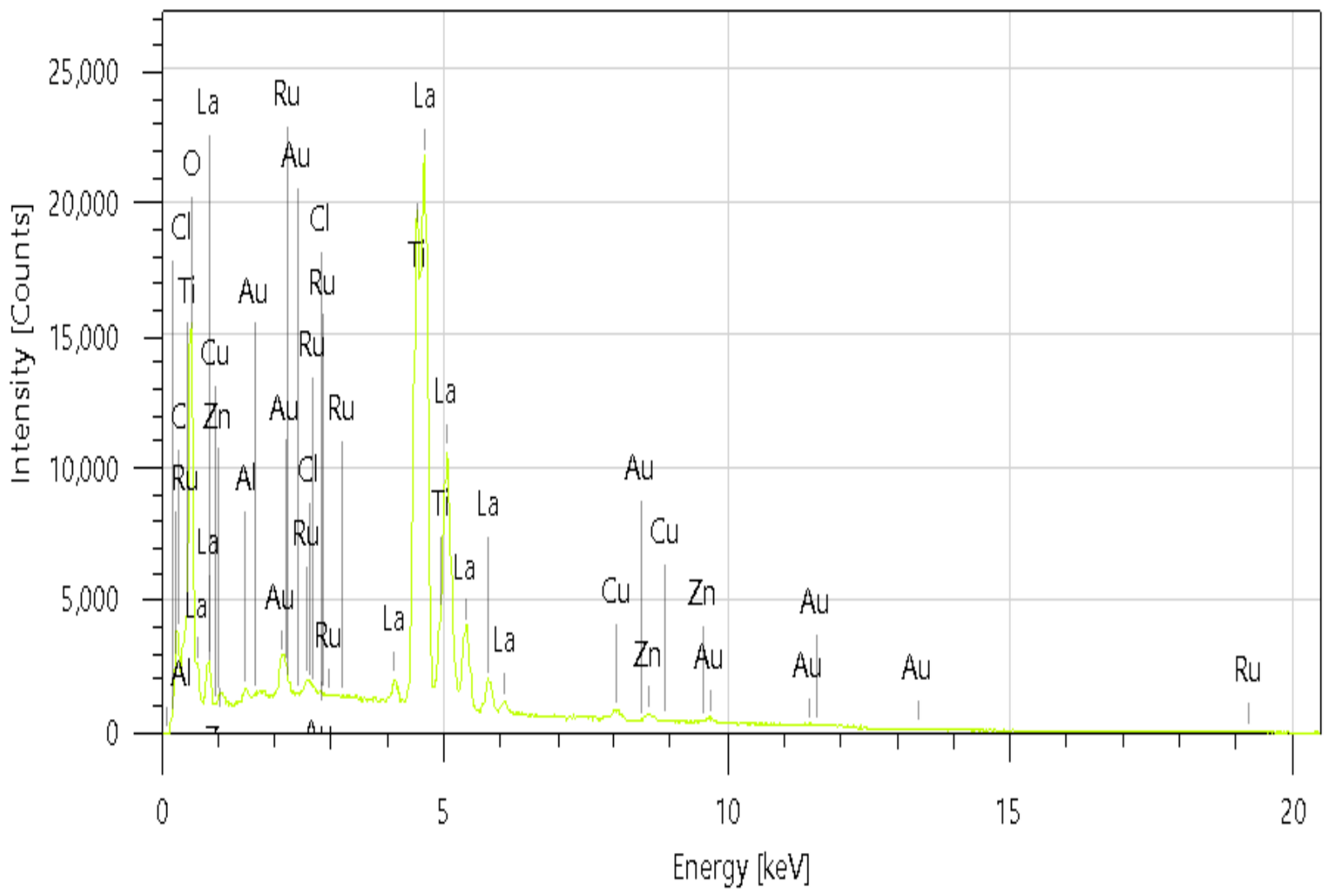

Figure S2: EDS(SEM) Spectrum of fresh catalyst (x=0.04).

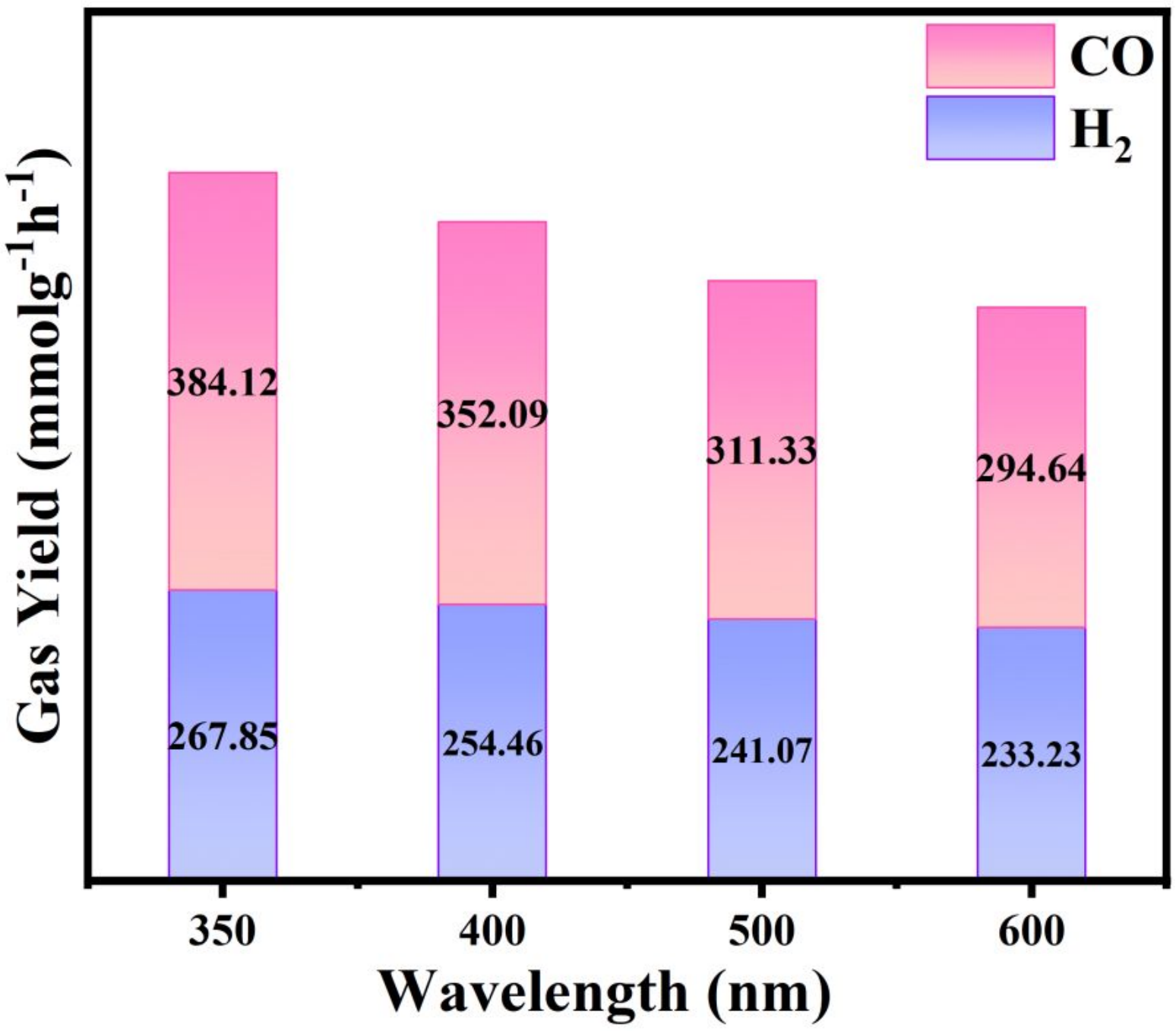

Figure S3: CO and H_2 yields at different wavelengths.

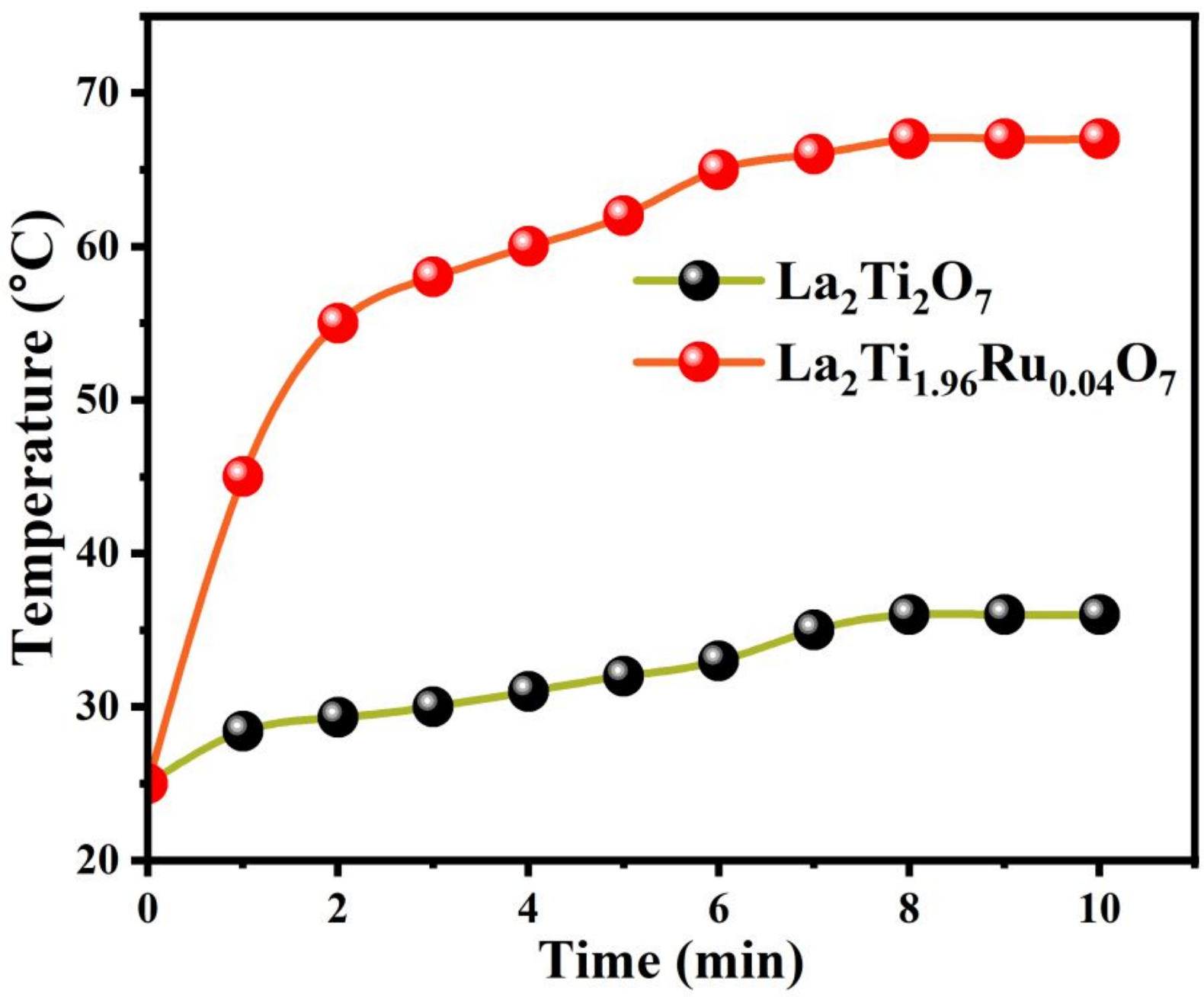

Figure S4: Light to temperature relationship with respect to time.

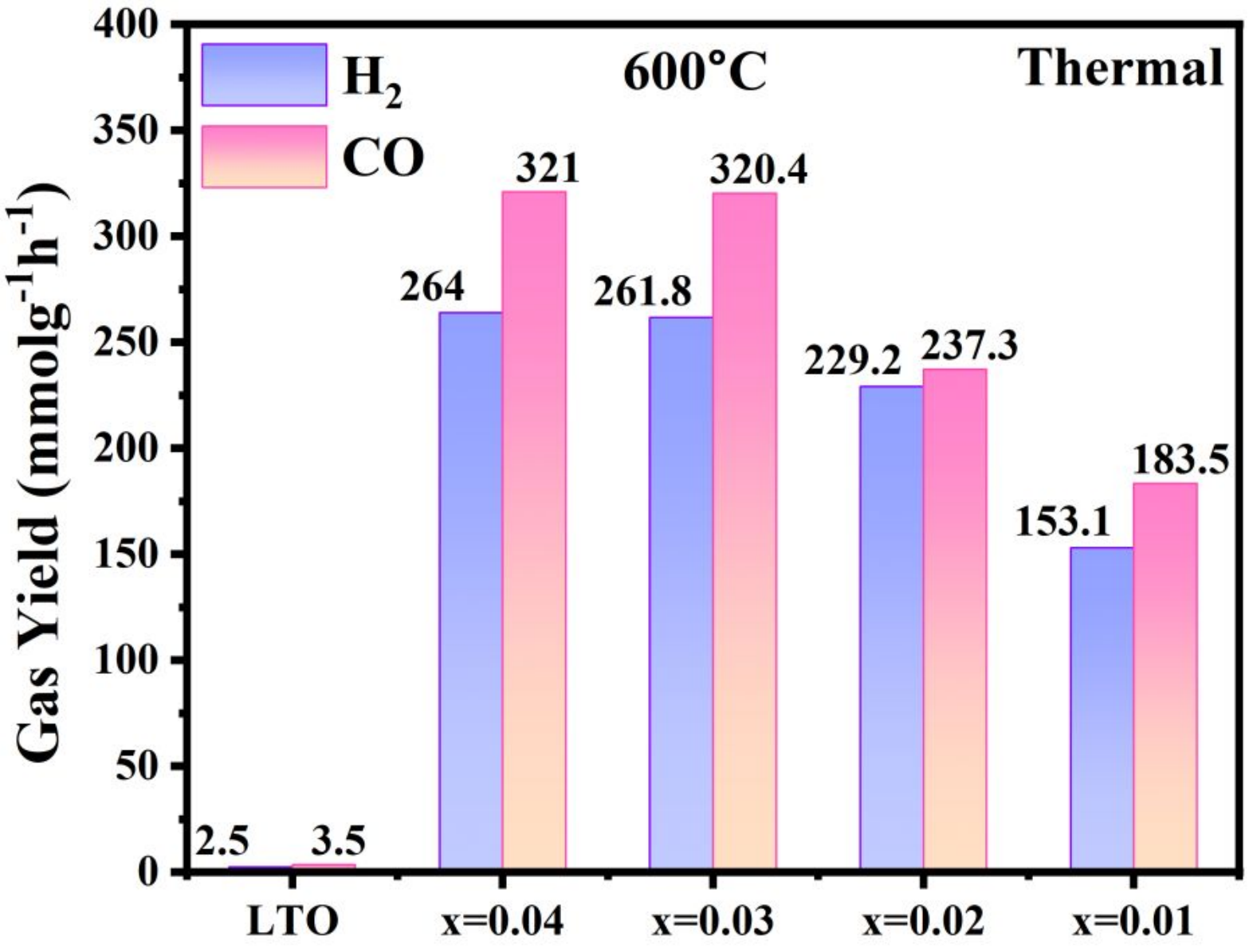

Figure S5: H_2 and CO yields of LTO, and x=0.01-0.04 at 600°C thermal conditions.

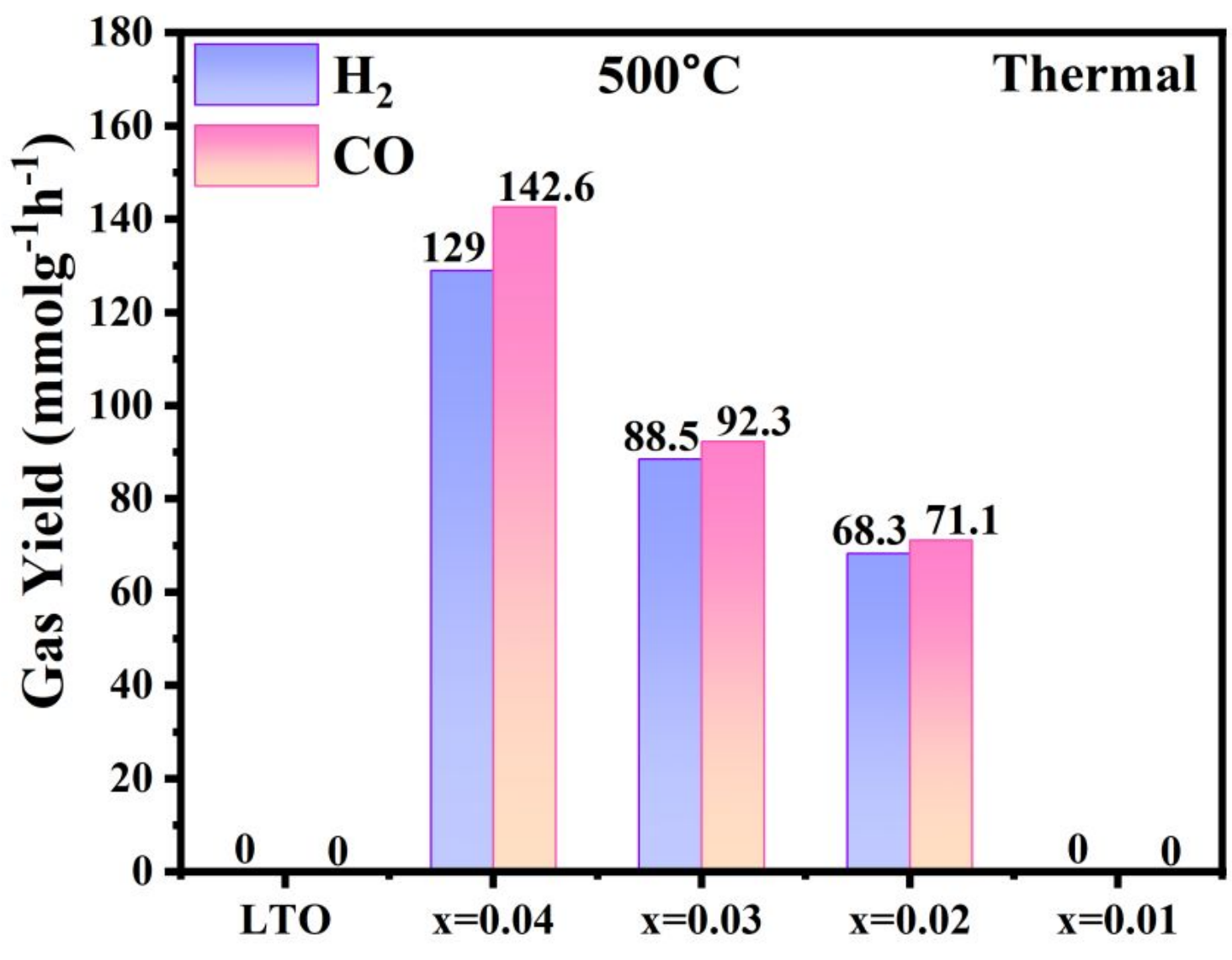

Figure S6: H_2 and CO yields of LTO, and x=0.01-0.04 at 500°C thermal conditions.

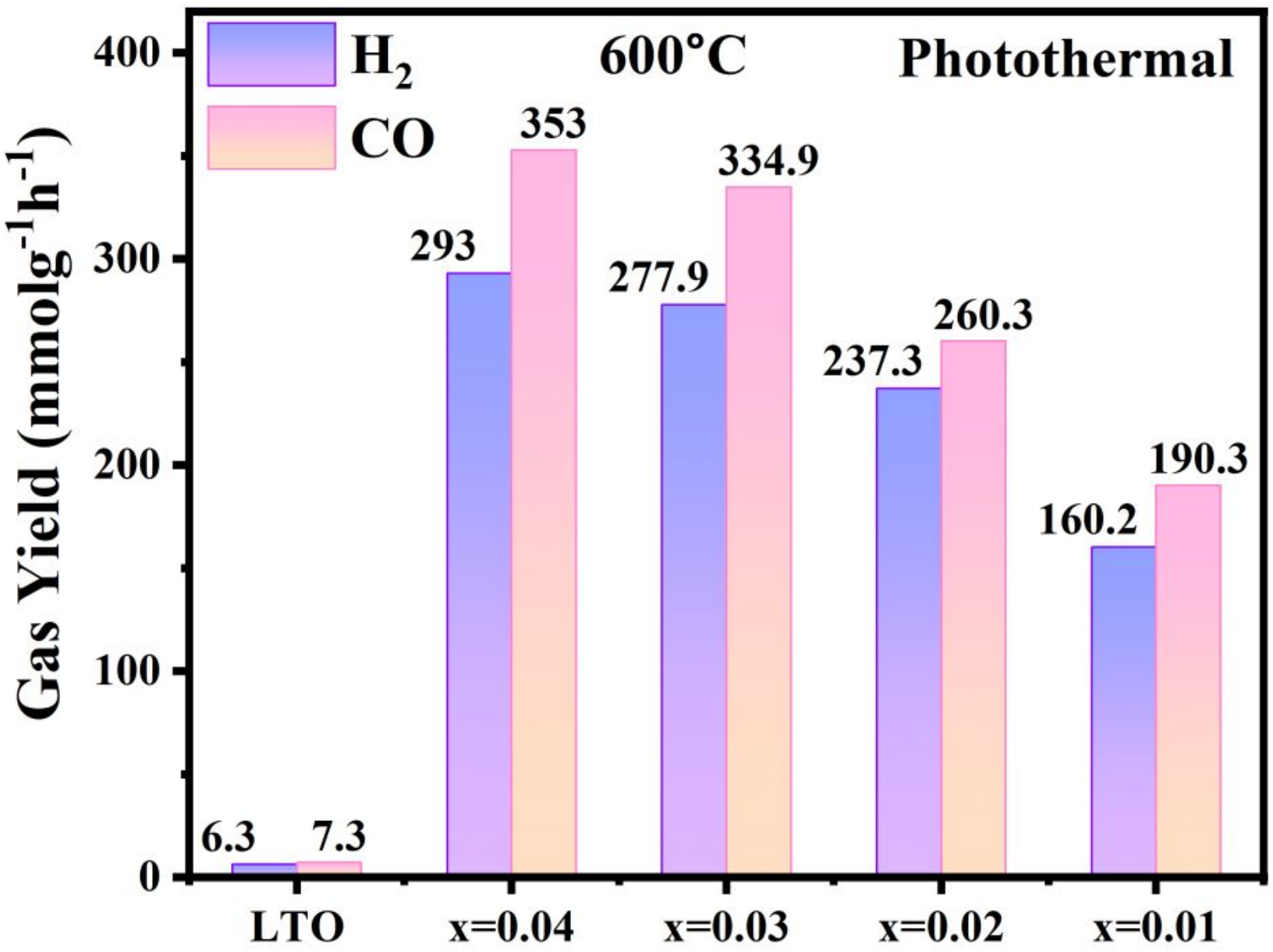

Figure S7: H_2 and CO yields of LTO, and x=0.01-0.04 at 600°C Photothermal conditions.

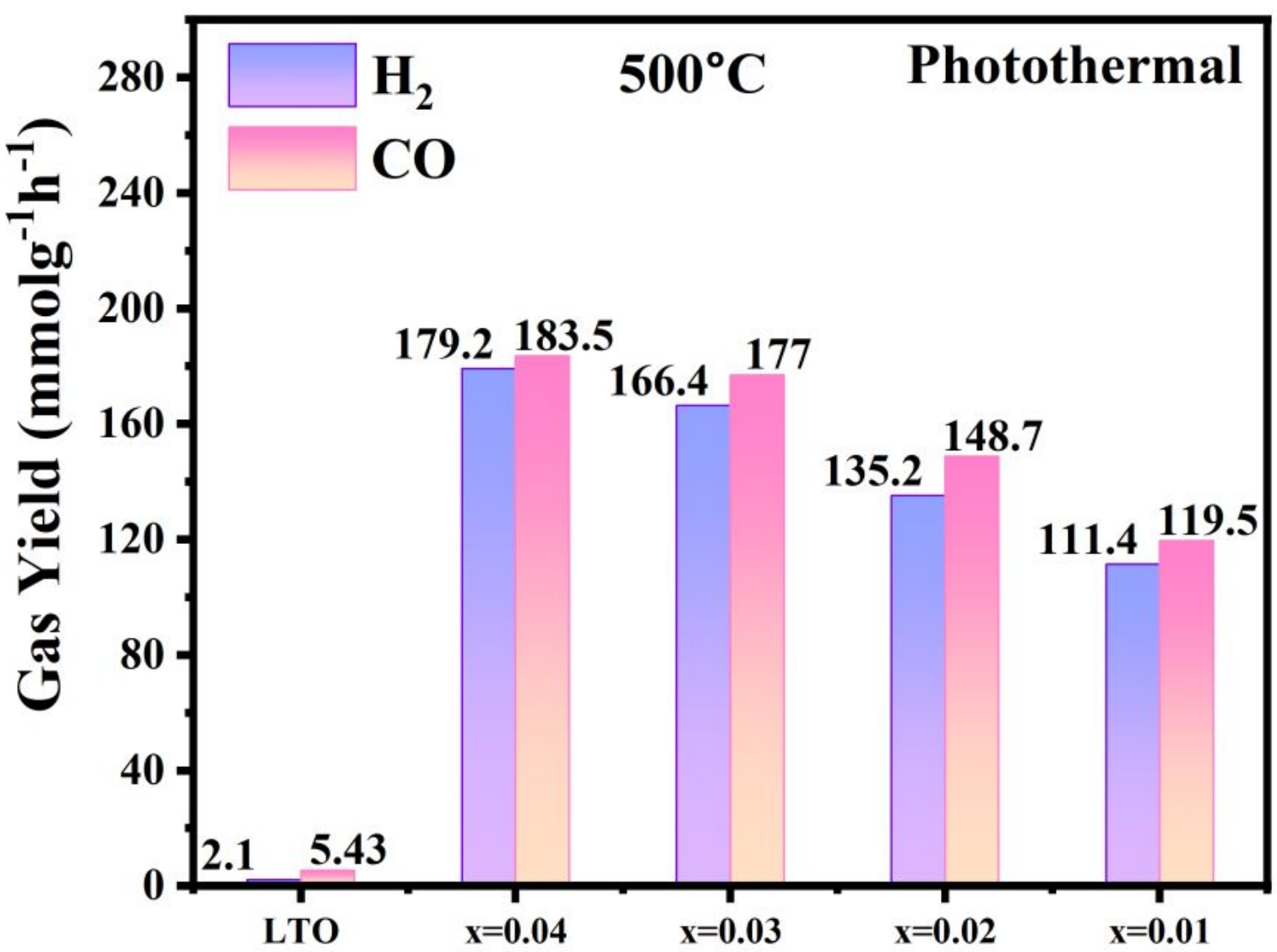

Figure S8: H_2 and CO yields of LTO, and x=0.01-0.04 at 500°C Photothermal conditions.

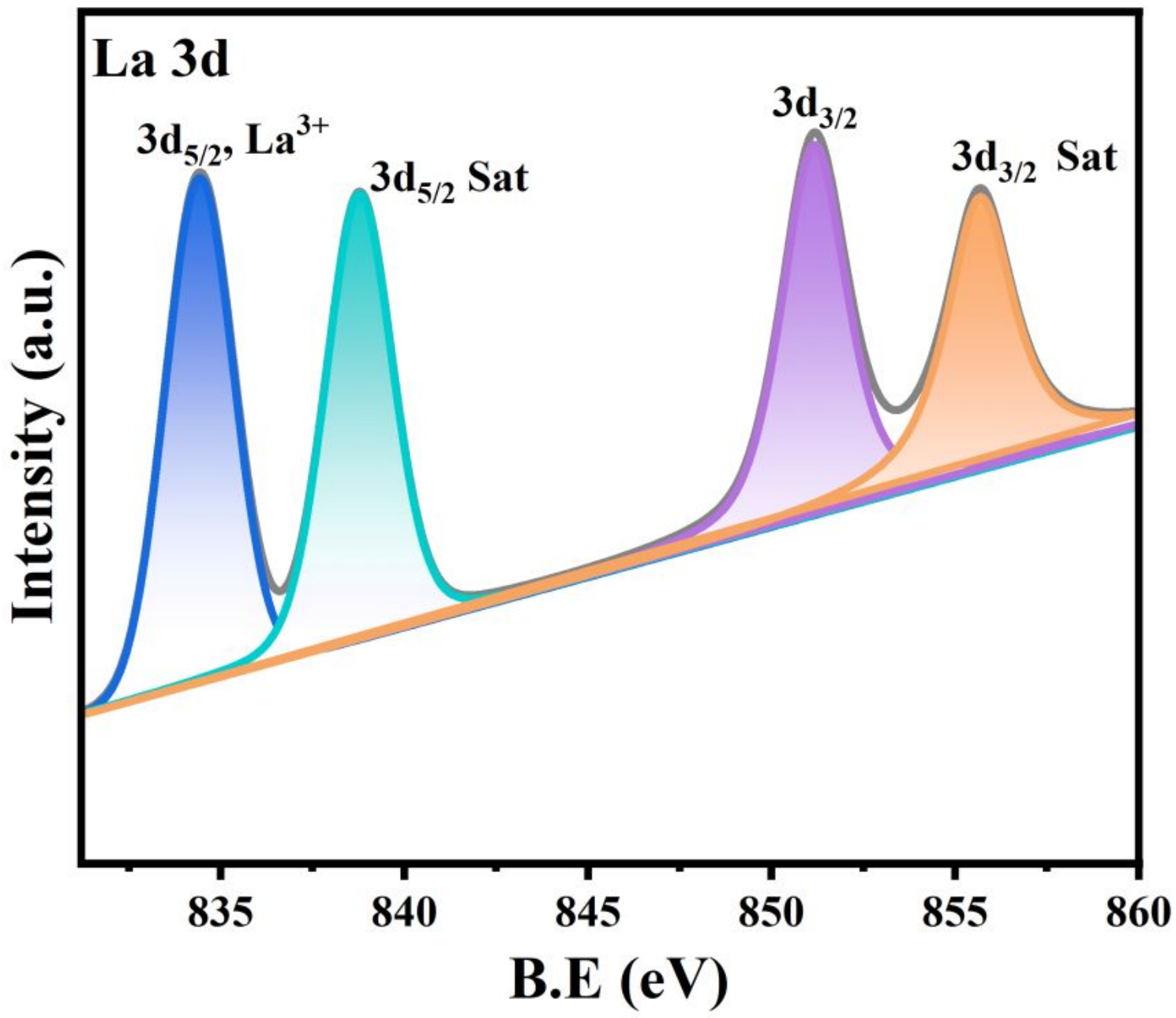

Figure S9: XPS analysis of La 3d of spent catalyst.

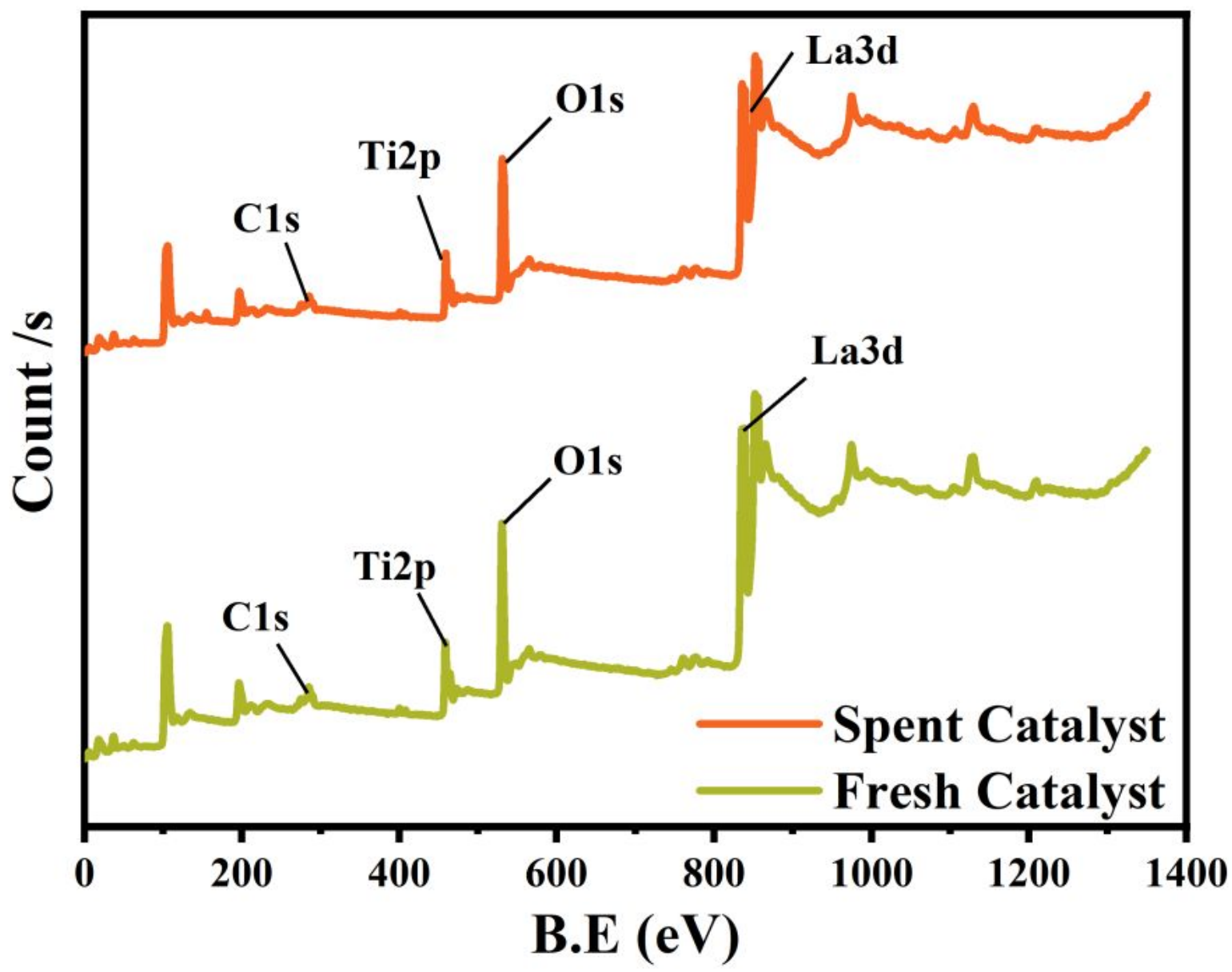

Figure S10: XPS survey of fresh and spent catalyst having La 3d, Ti 2p, O 1s and C 1s.

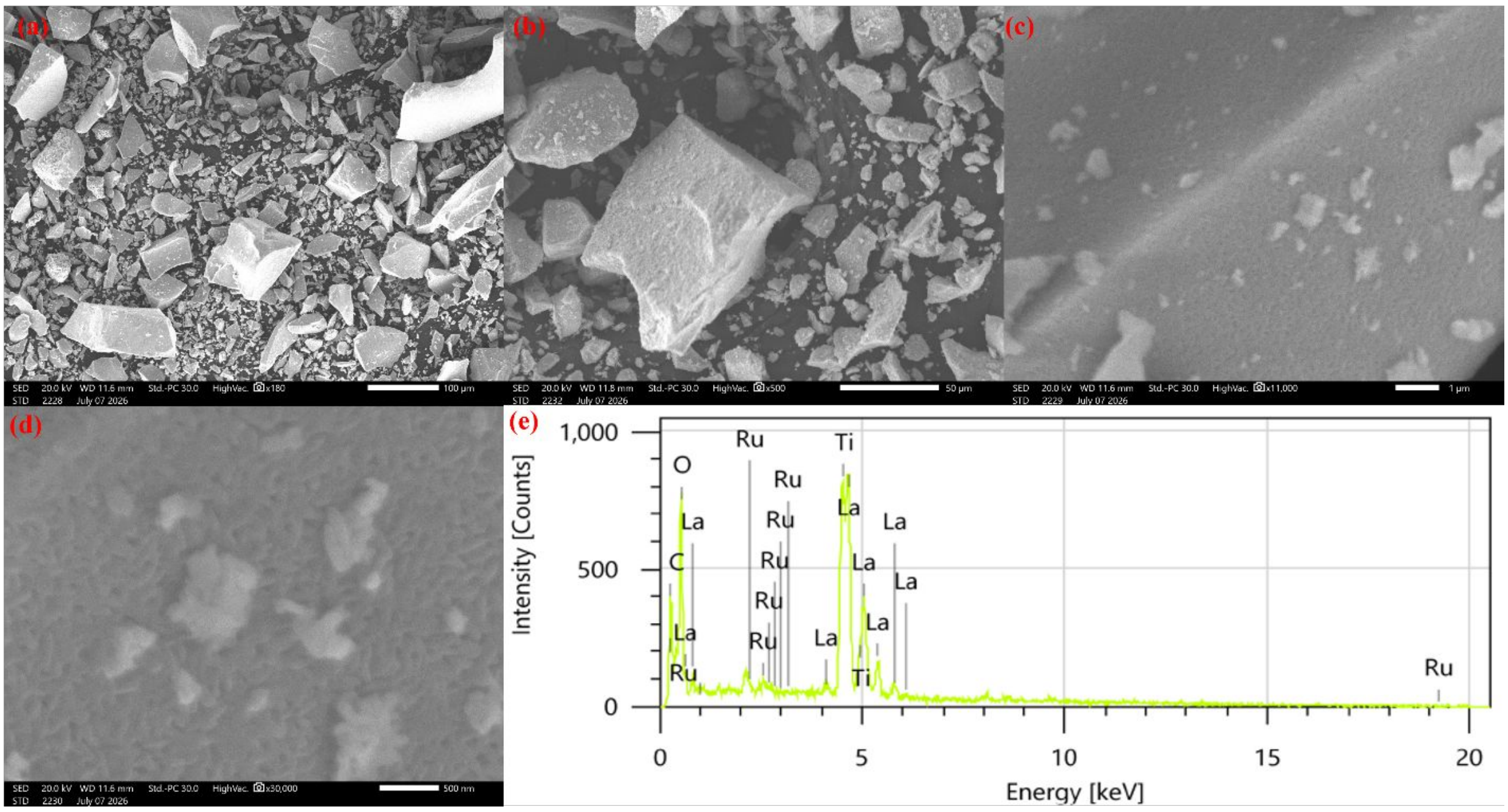

Figure S11: SEM and EDS images of spent catalyst x=0.04.

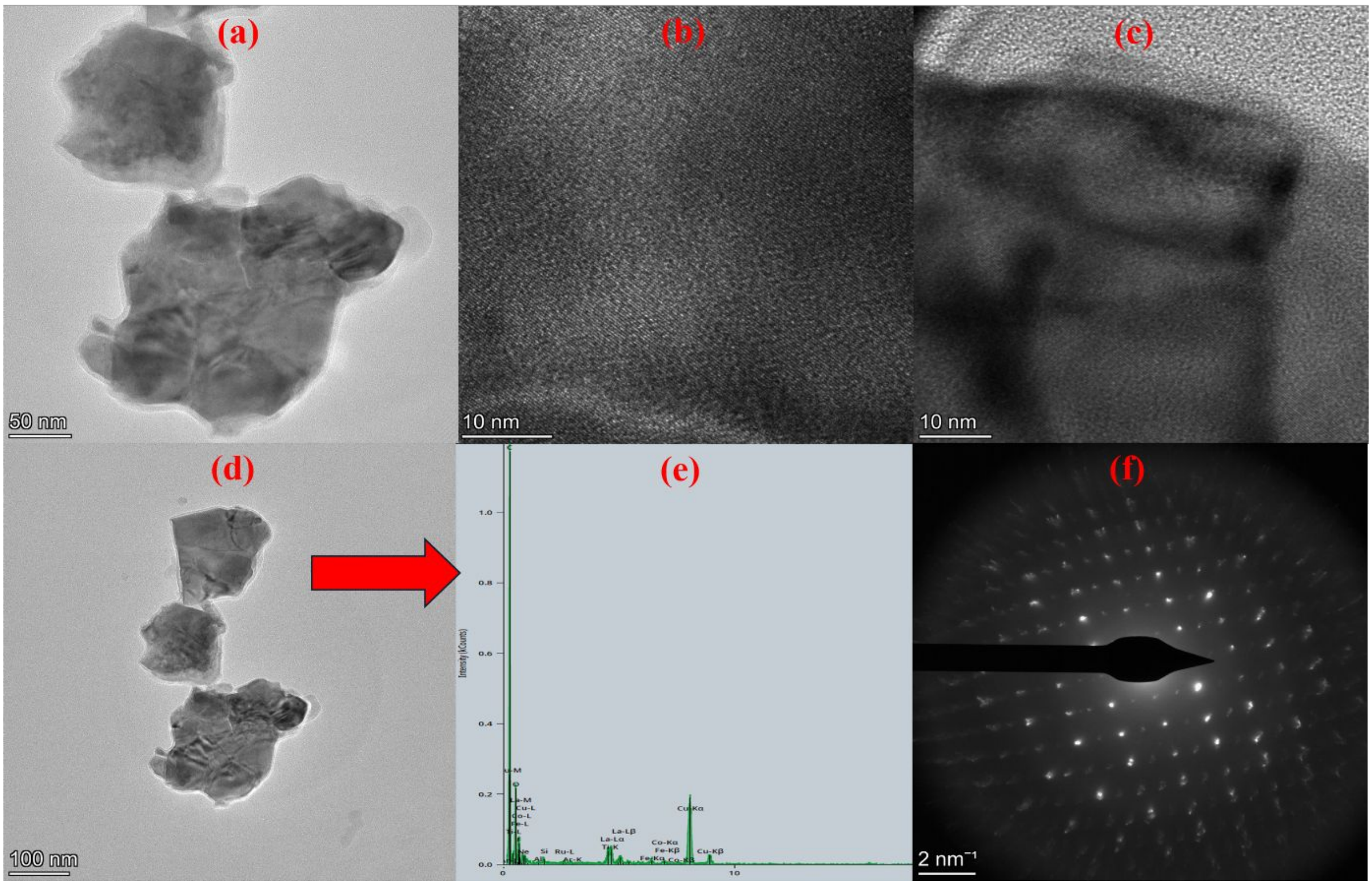

Figure S12: TEM images of spent catalyst (100h): (a-d), EDS: (e), SAED: (f).

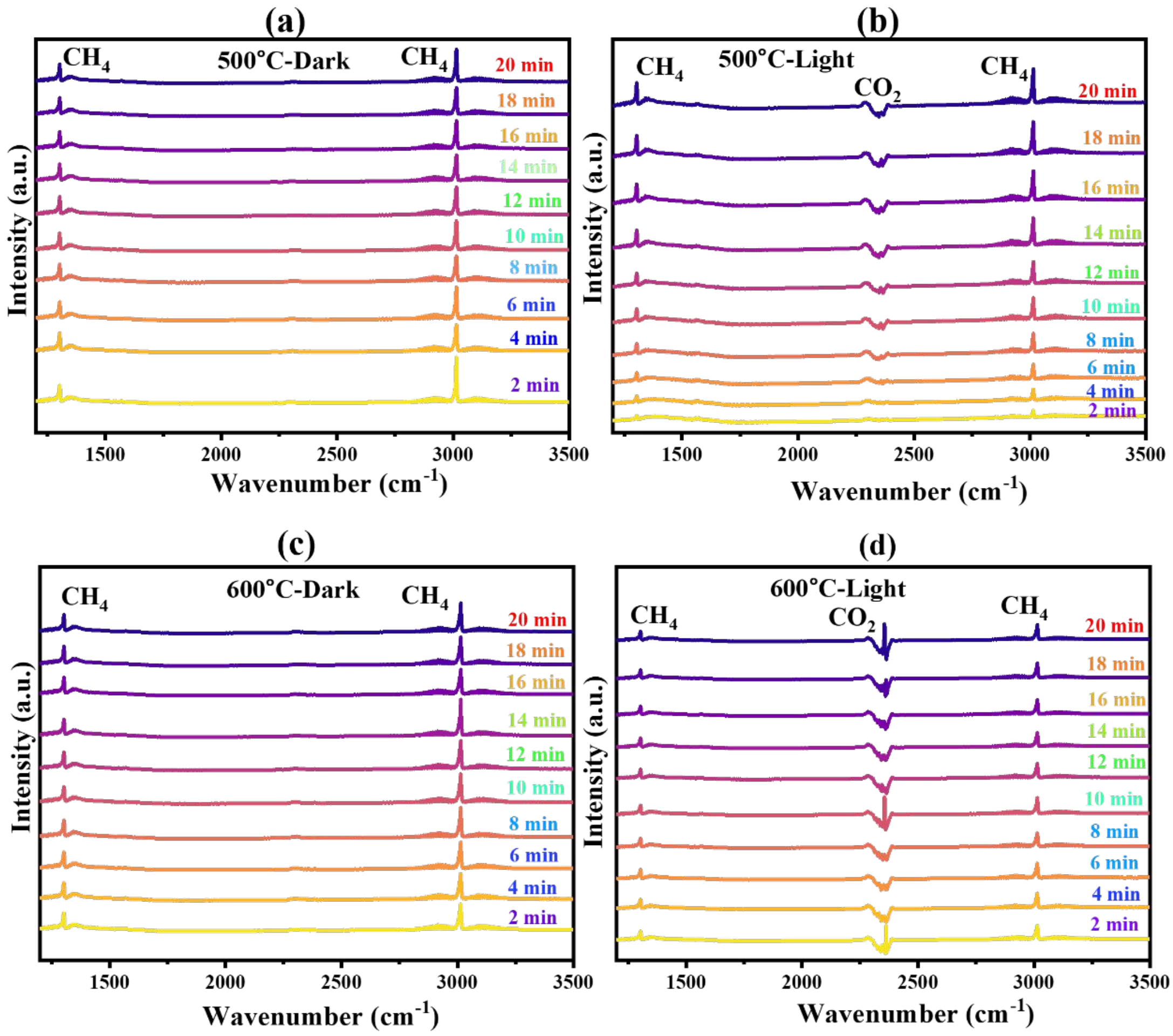

Figure S13: In-situ drifts with respect to time at 500°C and 600°C under light and dark.
